# Omentin expression in the ovarian follicles of Large White and Meishan sows during the oestrous cycle and *in vitro* effect of gonadotropins and steroids on its level: Role of ERK1/2 and PI3K signaling pathways

**DOI:** 10.1371/journal.pone.0297875

**Published:** 2024-02-26

**Authors:** Karolina Pich, Natalia Respekta, Patrycja Kurowska, Christelle Rame, Kamil Dobrzyń, Nina Smolińska, Joëlle Dupont, Agnieszka Rak

**Affiliations:** 1 Laboratory of Physiology and Toxicology of Reproduction, Institute of Zoology and Biomedical Research, Jagiellonian University in Krakow, Krakow, Poland; 2 Doctoral School of Exact and Natural Sciences, Jagiellonian University in Krakow, Krakow, Poland; 3 INRAE, UMR85, Unité Physiologie de la Reproduction et des Comportements, Nouzilly, France; 4 Department of Zoology, Faculty of Biology and Biotechnology, University of Warmia and Mazury in Olsztyn, Olsztyn-Kortowo, Poland; 5 Department of Animal Anatomy and Physiology, Faculty of Biology and Biotechnology, University of Warmia and Mazury in Olsztyn, Olsztyn-Kortowo, Poland; Alexandria University, EGYPT

## Abstract

Omentin (ITLN1) is a novel adipokine mainly expressed in the white adipose tissue. It plays a crucial role in the metabolic homeostasis and insulin sensitivity. Our last study documented that ITLN1 levels in the adipose tissue and plasma are lower in fat Meishan (MS) compared to normal weight Large White (LW) pigs. The aim of this study was to investigate transcript and protein concentrations of ITLN1 as well as its immunolocalisation in the ovarian follicles and examine the molecular mechanism involved in the regulation of its expression in response to gonadotropins (FSH, LH) and steroids (P_4_, T, E_2_). Ovarian follicles were collected from LW and MS sows on days 2–3, 10–12, and 14–16 of the oestrous. We found the elevated ITLN1 expression in the ovarian follicles and the increase of concentrations in follicular fluid (FF) of LW pigs *vs* MS pigs; in both breeds of pigs, the levels of ITLN1 increased with the oestrous progression. We noted ITLN1 signals in oocyte, granulosa and theca cells. Gonadotropins and steroids increased ITLN1 levels in the ovarian follicle cells of LW pigs, while in MS pigs, we observed only the stimulatory effect of LH and T. Both extracellular signal-regulated kinase (ERK1/2) and phosphatidylinositol 3′-kinase (PI3K) were involved in the regulation of ITLN1. Our study demonstrated the levels and regulation of ITLN1 in the porcine ovarian follicles through ERK1/2 and PI3K signaling pathways.

## Introduction

Omentin, also known as intelectin [1] and intestinal lactoferrin receptor [2], is a novel 34-kDa adipokine [3], first described in 1998 by Komiya *et al*. [4] in the intestinal Paneth cells of mice. The omentin gene is localized on a chromosomal region of 1q22–q23in humans and on chromosome 4 in pigs [5, 6]. A direct product of gene expression is a secretory protein consisting of 313 amino acids, including an 18-amino acid signal peptide [1], which occurs as two main structural forms in humans: omentin-1 (intelectin-1, ITLN1), a more active form presented in the circulatory system, and omentin-2, a homolog with 83% amino acid identity with ITLN1 [7], highly expressed in the small intestine and secreted into the lumen [5]. Moreover, in pigs, the complementary DNA sequence of this adipokine displays 82% and 79% similarity with the human and mouse homologs, respectively [8].

Gene and protein expression of ITLN1 has been noted in various tissues of humans, mice, and pigs. For example, in humans, ITLN1 was particularly confirmed in the visceral adipose tissue [9], thymus, spleen, heart [1], lungs [9], small and large intestines [5], and ovaries [10]. In mice, expression of ITLN1 was confirmed in the large intestine [1, 9], as well as in the lungs and brain [11], while in pigs, it is found in the peri renal adipose tissue [12, 13]. The specific receptors for ITLN1 have not yet been identified; nevertheless, some literature data report that ITLN1 increased the activity of insulin receptor substrate (IRS) by restraining the mammalian target of rapamycin [14]. Moreover, Cloix *et al*. [10] suggest that ITLN1 may modulate the phosphorylation of the insulin-like growth factor-1 (IGF-1) receptor beta subunit, as well as IRS-1, in the visfatin-knockdown human ovarian granulosa-like tumour (KGN) cells. Some studies indicate that dexamethasone, the agonist of the glucocorticoid receptor that promotes fat accumulation, stimulates the gene expression of ITLN1 in the human visceral adipose tissue [15]. Additionally, the gene and protein expression, as well as ITLN1 secretion are decreased by glucose and insulin in the human adipose tissue explants [16]. The studies also show that ITLN1 plasma levels are decreased in obese humans compared to normal weight controls [7], and ITLN1 gene expression in the peri renal adipose tissue is significantly lower in fat MS pigs compared to normal weight LW pigs [13]. Interestingly, the latest data suggest that ITLN1 enhances the sensitivity of adipose tissue to insulin and stimulates glucose metabolism, thus regulating the distribution of adipose tissue [16]. Moreover, ITLN1 increases glucose transport in the adipocytes *via* phosphatidylinositol 3′-kinase (PI3K) [7] and exhibits a proinflammatory role in the primary human adipocytes by the activation of extracellular signal-regulated kinase (ERK1/2) [17]. All of these data suggest that ITLN1 may regulate the distribution of adipose tissue and confirm its relationship with insulin resistance, which is often associated with obesity. Additionally, as suggested by the literature data, the metabolic status (negative energy balance as well as obesity) may impact on the reproductive functions of humans and animals by affecting the control of hypothalamus–pituitary–ovarian functions. These metabolic effects could lead to the hormonal homeostasis imbalance (e.g., increased luteinizing hormone [LH] and 17β-estradiol [E_2_] secretion as well as reducing circulating sex hormone binding protein concentrations) and, consequently, some disorders in the ovulation, implantation, oocyte and embryo development, or pregnancy loss [18].

The studies about the expression and role of ITLN1 in the reproduction system are limited. Cloix *et al*. [10] showed the gene and protein expression of ITLN1 in the human granulosa-lutein cells (hGLC) in the healthy and polycystic ovary syndrome (PCOS) patients and in KGN cells. Moreover, the authors assessed that PCOS women have decreased ITLN1 levels in the follicular fluid (FF) compared to the control, healthy patients [10], suggesting its dependence on the hormonal status; PCOS is associated with the elevated circulating levels of LH and hyperandrogenism [19]. Moreover, the concentrations of circulating ITLN1 in the blood negatively correlates with the concentrations of E_2_ [16] and free testosterone (T) [20], suggesting a possible involvement of sex steroids in regulating ITLN1 production. Only one study shows that hormones like insulin, IGF-1, and FSH increase protein expression of ITLN1 in hGLC and KGN cell line [10].

Thus, we hypothesize that expression of ITLN1 in the porcine ovary is dependent on phase of the oestrous cycle, and its level is regulated by different reproduction-related hormones. This study aimed to investigate the gene and protein expression of ITLN1 in the ovarian follicles, its immunolocalization, and its concentrations in FF in normal weight LW and fat MS sows during the oestrous. In the next step, we assessed the *in vitro* effect of gonadotropins (FSH and LH) and steroids (P_4_, T, and E_2_) on ITLN1 protein expression in the ovarian follicular cells of both LW and MS sows. Additionally, we investigated the involvement of ERK1/2 and PI3K on the regulation of ITLN1 levels in ovarian cells. As reported by literature data, MS sows reach puberty at an earlier age [21], and they are fatter than LW sows [22]. Also, MS pigs have a greater intramuscular fat content, backfat thickness and the muscles of MS pigs generally contain higher amount of lipids than that of LW pigs [23] and this is also observed in obese people [24]. MS pigs similar as obese people tend to have a higher triglyceride concentration as well as HOMA-IR score [25] or lower blood urea nitrogen level [26]. Our previously study confirmed that the adipokines profile in plasma and adipose tissue in fat MS pigs was similar as in obese human; concentration of leptin was increased, while adiponectin was decreased [13]. Interesting, MS pigs characterize some many reproductive diseases similar to note in obese female including fewer cells in the trophectoderm [27] or a reduction in embryo developmental rate [28]. However, obese women have fertility problems, while MS sows are very fertile. The LW breed name also Yorkshire is one of the most popular commercial pig breed for successful pig farming [29] as well as are a great model to study reproduction because of similarities in the reproductive track anatomy, cycle length (around 21 days in pigs and 28 days in women), hormonal fluctuations during oestrous and menstrual cycle as well as similarities in fertilization, *in vitro* development to the blastocyst stage, pregnancy and puberty [30–32].

## Materials and methods

### Ethical issue

The use of sows was in accordance with directive 2010/63/EU of the European Parliament and of the Council of 22 September 2010 on the protection of animals used for scientific purposes. Ovarian follicles have been collected during meat processing as abattoir by-products by highly qualified and experienced laboratory staff. Thus, according to the ethical issues for the protection of animals, this project does not require the consent of the competent ethics committee for animal experiments.

### Animals and ovaries collection

The research was carried out on the ovarian follicles collected from normal weight LW and fat MS sexually mature gilts at a local abattoir under veterinarian control. LW gilts, with an average weight of 91.76 ± 8.2 kg and age 214.7 ± 8.2 days, and the same number of MS gilts, with an average body weight of 30.62 ± 5.8 kg and age 179.0 ± 6.1 days, were used in this study. The zootechnical parameters (body weight, backfat thickness, age at the puberty) were routinely collected by the UEPAO (doi: https://doi.org//10.15454/1.5573896321728955E12) and pig phenotyping as well as Innovative breeding facility (doi: https://doi.org//10.15454/1.5572415481185847E12) experimental units for the monitoring of the breeding. The MS pigs are genetically able to faster fattening than LW, and fattening does not depend on diet as well as slaughter time [33]. Moreover, as the literature data suggest obese pigs, are often marked by excessive backfat (24.3 ± 0.4 mm in MS compared to 16.6 ± 0.3 mm in LW pigs) [13]. Additionally, the biochemical parameters as well as the pattern of adipokines concentration in plasma and expression in perirenal adipose tissues from MS pigs [13, 25] are similar to those reported in humans [34, 35]. Also, MS pigs reach puberty earlier (86.1 ± 1.30 days) than LW pigs (171.96 ± 2.8 days) [13]. In this way, they are a good animal model for studying the effect of obesity on reproduction, because also childhood obesity has been associated with the earlier onset of puberty and menarche [36]. The reduced number of cells present in fat MS embryos results from a selective reduction in the number of trophectoderm [37] and also blastocysts collected from obese women contain fewer cells notably in the trophectoderm [27]. However, the prolificacy of the MS breed is about three to four piglets greater than in LW [38].

Within a few minutes after slaughter, the ovaries were removed and placed in 0.1 M ice‐cold phosphate‐buffered saline (PBS, S1 Table) supplemented with antibiotics and transported to the laboratory within 30 min of collection. Based on the morphological examination of the ovaries [39], ands levels of P_4_ and E_2_ in plasma as well as E_2_ in the FF (S3 Table) during oestrous cycle in LW and MS pigs (S1 Fig) we collected small follicles (SF; 2–4 mm in diameter; days 2–3 of oestrous), medium follicles (MF; 4–6 mm in diameter; days 10–12 of oestrous), and large follicles (LF; 8–12 mm in diameter; days 14–16 of oestrous), as well as FF on days 2–3, 10–12, and 14–16 of the oestrous cycle from LW and MS pigs. In pigs, day 1 of estrous cycle refers to the first day after ovulation [40].

**Fig 1:**
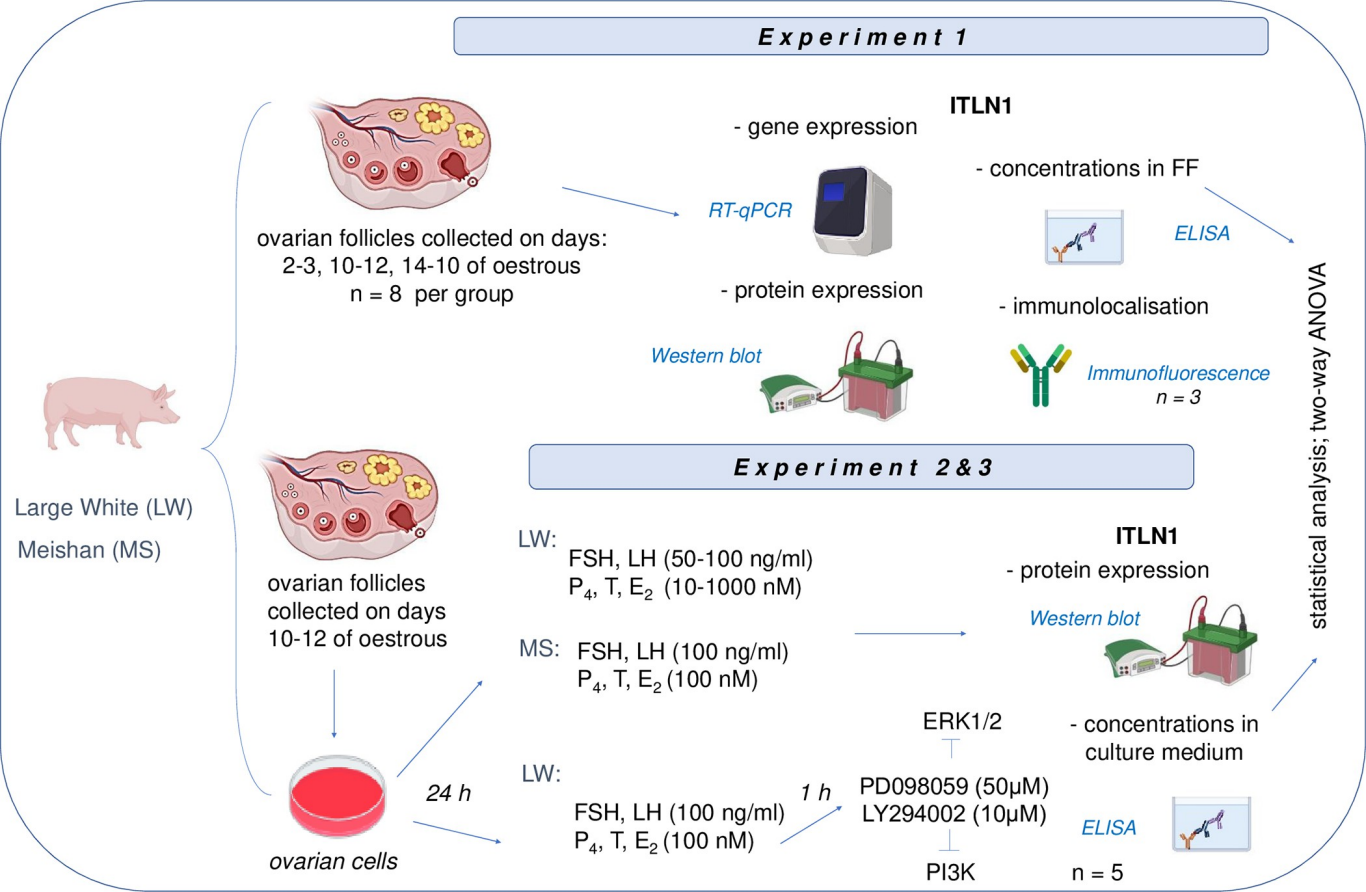
Schematic diagram of materials and methods used for assessing the gene and protein expression of ITLN1, its immunolocalization in the ovarian follicle cells, concentrations in FF from LW and MS pigs during oestrous cycle as wells the effect of gonadotropins and steroids on ITLN1 level in ovarian follicle cells and its secretion to the culture medium in LW and MS pigs with involvement of ERK1/2 and PI3K in the regulation of ITLN1 expression in LW pigs. ANOVA, analysis of variance; E_2_, 17β-estradiol; ELISA, enzyme-linked immunoassay; ERK1/2, extracellular signal-regulated kinase; FF, follicular fluid; FSH, follicle-stimulating hormone; Gc, granulosa cells; ITLN1, intelectin 1; LH, luteinising hormone; LW, Large White; MS, Meishan; P_4_, progesterone; PI3K, phosphatidylinositol 3′-kinase; RT-qPCR, reverse transcription quantitative real-time polymerase chain reaction; T, testosterone; Th, theca interna cells.

In *Experiment 1*, to determine ITLN1 gene and protein expression, whole healthy ovarian follicles with no atretic changes of LW and MS pigs were immediately frozen in liquid nitrogen and stored at -70°C (the number of animals per each phase of the oestrous cycle were 8 for LW and 8 for MS, n = 8). Additionally, the whole ovaries from LW pigs were collected during the oestrous (days 2–3, 10–12, and 14–16; the number of animals per each phase of the oestrous cycle were 3 for LW, n = 3) and immersed in 4% buffered paraformaldehyde for future immunofluorescence analysis (see Experimental protocol, Fig 1). To assess ITLN1 concentrations in FF, the samples were prepared by low-speed centrifugation (2,000 g at 4°C for 10 min) and stored at -20°C to determine the ITLN1 level.

### RNA isolation and real-time polymerase chain reaction (qRT-PCR)

Total RNA was extracted using QIAzol Lysis Reagent (S1 Table), as previously described [13]. All methods were performed according to the manufacturer’s protocols. RNA quantity and purity (A260/A280) were determined using a spectrophotometer (DeNovix, United States). The total RNA of follicles cells was reverse-transcribed for 1 h at 37°C in a final reaction volume of 20 μl as previously [13]. cDNAs were amplified in the presence of specific primers for ITLN1 and reference genes (Cyclophylin A—PPIA; β-actin—ACTB; glyceraldehyde-3-phosphate dehydrogenase—GAPDH). The primers for the examined genes were designed based on the gene sequences deposited in GenBank. The reaction mixtures at the final volume of 20 μl consisted of 10 μl iQ SYBR Green Supermix, 0.25 μl of each primer (10 μM), 4.5 μl of water, and 5.0 μl of the template. The cDNA templates were amplified and detected using the MYIQ Cycler real-time PCR system following the protocol previously described [10, 13]. The descriptions of the different primers are as follows: ITLN1 (F: 5′-GATTCTGCCTCCTGCTGTTC-3′ and R: 5′-ATACAGGCCATCACCTGCTC-3′), PPIA (F: 5′-GCATACAGGTCCTGGCATCT-3′ and R: 5′-TGTCCACATGCAGC AATGGT-3′), ACTB (F: 5′-ACGGAACCACAGTTTATCATC-3′ and R: 5′-GTCCCAGT CTTCAACTATACC-3′), and GAPDH (F: 5′-GCACCGTCAAGGCTGAGAAC-3′ and R: 5′-ATGGTGGTGAAGACGCCAGT-3′). Relative gene expression levels of examined genes were determined using the comparative cycle threshold 2^−ΔΔCt^ method [41], and normalization by the geometrical mean of the reference gene’s expression levels was performed.

### Western blot

Western blot was performed as described by Rak *et al*. [42, 43]. Ovarian follicles were homogenised on ice with a cold buffer and then centrifugation at 15,000 g at 4°C for 30 min, and the protein concentrations was determined by the Bradford method (BioRad) using bovine serum albumin (BSA) as a standard. Equal amounts of protein lysates (~30 μg of protein/lane) were separated by hand-casting 10% polyacrylamide gels and transferred to polyvinylidene fluoride membranes. The blots were blocked with Tris‐buffered saline containing 0.1% Tween 20 (TBST) and 5% BSA for 1 h. Then, the membranes were incubated overnight at 4°C with the anti-ITLN antibody (sc-130923, Santa Cruz Biotechnology, United States; monoclonal; host species: mouse) diluted at 1:500 in 5% BSA/TBST and ACTB antibodies diluted at 1:1000 in 5% BSA/TBST. Next, the membranes were washed with TBST and incubated for 1 h with horseradish peroxidase (HRP)-conjugated antibody (7076S, Cell Signaling Technology, United States; monoclonal; host species: horse) diluted at 1:1000. An ACTB protein was used as a loading control and as a reference protein to determine relative amounts of analysed proteins. Immunolabeled bands were detected by chemiluminescence using a chemiluminescent HRP substrate reagent. Membranes were visualized using the ChemiDoc™ Imaging System (BioRad). All visible bands were quantified using ImageJ analysis software (National Institute of Health). The results were expressed as the intensity signal in arbitrary optical density units.

### Immunohistochemistry

Tissue preparation for fluorescent immunostaining was conducted as a standard procedure described by Rytelewska *et al*. [44]. In brief, prepared tissue slides of the ovaries from LW pigs were incubated were incubated overnight with rabbit polyclonal antibodies against ITLN1 (dilution 1:100), rinsed in PBS three times, and incubated with the secondary antibody (1:1000, A28175, Invitrogen, United States) for 90 min at room temperature. After that, tissue sections were rinsed in PBS three times and again dehydrated. Finally, the sections were covered with histology mounting medium Fluoroshield ™ with DAPI for nuclear counterstaining. The labelled tissue sections were analyzed with an Olympus BX51 research microscope (Olympus, Japan) equipped with an EXFO x‐Cite Series 120Q fluorescence illuminator (Excelitas Technologies Corp, United States) using appropriate filters set for DAPI and Alexa Fluor ® 488. Images were acquired with an Olympus DP72 microscope digital camera and Cell F software (Olympus, Japan). For the negative control, the primary antibody was omitted and tissues were incubated in 0.1 M PBS.

### Enzyme-linked immunosorbent assay (ELISA)

The concentrations of ITLN1 in FF and culture medium from LW and MS pigs were determined using a commercial ELISA kit for porcine ITLN1 (E07O0010, Bluegene, China) according to the manufacturer’s protocol. The range of the standard curve was 0–100 ng/ml. The sensitivity of the assay was defined as 0.1 ng/ml. Absorbance values were measured at 450 nm using a Varioskan™ LUX multimode microplate reader (ThermoFisher, United States). The intra‐assay coefficient of variation of the assay was 5.2%.

### Cell isolation and *in vitro* cell culture

The procedure of porcine granulosa (Gc) and theca (Th) cells isolation and *in vitro* culture was conducted as described by Stoklosowa *et al*. [45] with modifications. To in vitro experiments we decided used MF on days 10–12 of the oestrous cycle from LW and MS pigs (the number of animals were 5 for LW and 5 for MS, n = 5). Briefly, Gc was scrubbed mechanically, while Th layers from the same follicles were exposed three times to 0.25% trypsin in PBS for 10 min at 37°C. Following isolation, cells were counted using a Bürker chamber, and cell viability was determined by 0.4% trypan blue. The mean viability of Gc and Th was 94 ± 1.5% and 90 ± 1%, respectively. Cells were cultured in 96-well culture plates at the final concentrations of 5 x 10^4^ (Gc: 4 x 10^4^ and Th: 1 x 10^4^, reflecting that observed *in vivo* in the ovary) in M199 medium supplemented with 10% fetal bovine serum (FBS). All cultures were maintained in a humidified incubator (terms: 5% CO_2_/95% O_2_; 37°C).

### Experiment 2

To study the effect of hormones involved in the oestrous cycle progression on the ITLN1 level, ovarian cells were isolated from MF on days 10–12 of the oestrous (phase of the oestrous chosen based on ITLN1 expression results) from LW and MS pigs and incubated for 48 h in M199 supplemented with 1% FBS as a control medium or with FSH or LH at the doses of 50–150 ng/ml for LW or 100 ng/ml for MS and steroids P_4_, T, or E_2_ at the doses of 10–1000 nM for LW or 100 nM for MS. Cell treatments for each tested factor and dose were performed in five wells of culture plate. Doses of hormones in LW pigs were chosen based on our previous study and according to the concentrations in FF [38, 42], while in MS pigs, they were based on the results obtained for LW pigs. After incubation, the medium was stored at -20°C to assess ITLN1 concentrations by ELISA, while the cells were boiled in Laemmli buffer for 5 min then stored at -20°C for ITLN1 protein expression analysis by Western blot. Five independent *in vitro* cultures were performed (see Experimental protocol, Fig 1).

### Experiment 3

To investigate the involvement of ERK1/2 and PI3K on ITLN1 levels, cells isolated from the MF on days 10–12 of the oestrous cycle were plated in 96-well culture plates in M199 medium with 10% FBS for 24 h. Next, the medium was changed to 1% FBS, and the cells were pre-treated for 1 h with the ERK1/2 inhibitor (PD098059) the dose of 50 μM and the PI3K inhibitor (LY294002) at a dose of 1 μM. Then, gonadotropins, at the dose of 100 ng/ml, and steroids, at the dose of 100 nM, were added for an additional 48 h of culture. The concentrations of the inhibitors were chosen based on previous data [42, 46]. Previous data demonstrated inhibitory effect of PD098059 on phosphorylation of ERK1/2 signaling in rat granulosa cells [47, 48]. After incubation, the medium was stored at -20°C for ITLN1 concentrations, while the cells were boiled in Laemmli buffer for 5 min then stored at -20°C for ITLN1 protein expression analysis. Five independent *in vitro* cultures were performed (see Experimental protocol, Fig 1).

### Statistical analysis

Statistical analysis was performed in the GraphPad Software (PRISM software version 5). To determine the differences in ITLN1 gene and protein expression as well as its concentration in the FF during oestrous cycle between two tested types of breeds we performed two-way ANOVA test. Different lower case letters indicate statistical significance between days of oestrous cycle (2–3, 10–12, and 14–16) in one tested breed (LW or MS), whereas different capital letters indicate a significant difference between types of pig breeds.

To demonstrate the differences in the effect of gonadotropins as well as steroid hormones on ITLN1 protein expression as well as its concentration in the culture medium in LW or MS pigs we performed one-way ANOVA test as well as two-way ANOVA to compare the effect of FSH (100 ng/ml), LH (100 ng/ml) as well as P_4_, T, and E_2_ (100 nM) on ITLN1 levels between two tested breeds of pigs. Different lower case letters indicate statistical significance between studied hormones in LW or MS, while different capital letters indicate a significant difference between types of pig breeds. To indicate the involvement of ERK1/2 as well as PI3K signaling pathways in the regulation of protein expression of ITLN1 as well as its concentration in culture medium in LW pigs we performed one-way ANOVA test. Different lower case letters indicate statistically significant difference.

In each case, ANOVA analysis was followed by Tukey’s honest significant post hoc test. All data were also tested for the assumptions of normality (Shapiro–Wilk test) and homogeneity of variances (Levene’s test). Results were reported as mean ± standard error of the mean. Statistical significance is indicated by different letters (p < 0.05).

## Results

### Expression of ITLN1 in the ovarian follicles from LW and MS pigs and its concentrations in FF

**Fig 2:**
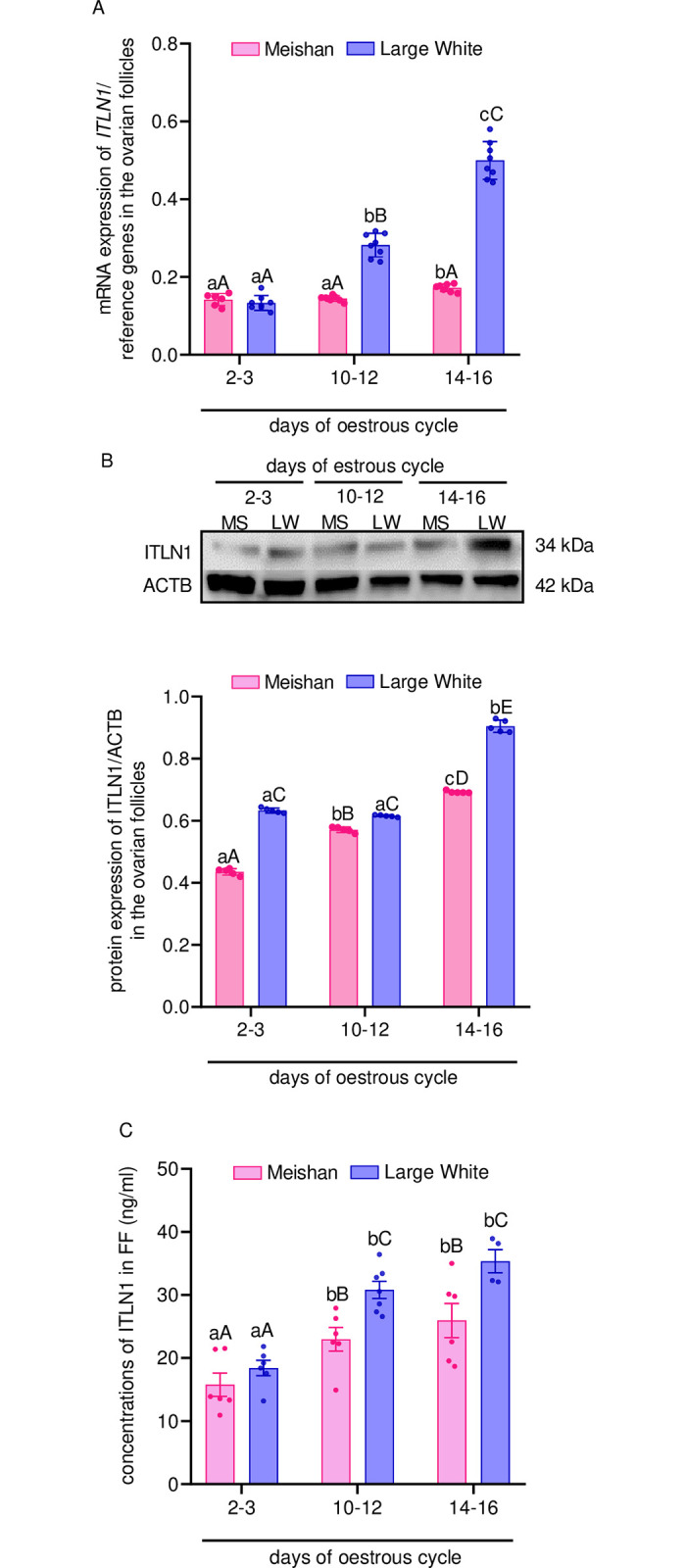
Gene (A) and protein (B) expression of ITLN1 in the ovarian follicles, as well as ITLN1 concentrations (C) in the FF from LW and MS pigs during the oestrous cycle (days: 2–3, 10–12, 14–16). Relative gene expression was determined using RT-qPCR. Results were normalised with the geometric mean of reference gene (GAPDH, PPIA, ACTB) expression using comparative cycle threshold method. The protein expression was detected by western blot assay; each protein abundance was evaluated densitometrically and expressed as the ratio relative to ACTB abundance. ITLN1 concentration was determined by ELISA assay. Results were reported as the mean ± SEM of eight (gene and protein expression of ITLN1) or five (ITLN1 concentrations in the FF) independent determinations. The lower case letter denotes differences between days of oestrous cycle whereas an upper case letter denotes differences between types of pig breeds (p < 0.05; two-way ANOVA followed by Tukey’s HSD test). ACTB, actin beta; ANOVA, analysis of variance; ELISA, enzyme-linked immunosorbent assay; FF, follicular fluid; SEM, standard error of the mean; GAPDH, glyceraldehyde-3-phosphate dehydrogenase; LW, Large White; MS, Meishan; PPIA, peptidylprolyl isomerase A; Tukey’s HSD, Tukey’s honest significant difference; RT-qPCR, reverse transcription quantitative real-time polymerase chain reaction.

We noted that gene and protein expression in the ovarian follicles from LW and MS pigs increased along with oestrous cycle progression; elevated gene and protein levels were observed on days 10–12, as well as days 14–16, of the oestrous of LW and MS. Moreover, comparing both gene and protein expression of ITLN1, we observed higher gene and protein expression of ITLN1 in the ovarian follicles from LW pigs compared with MS pigs in all studied days of the oestrous (p < 0.05; Fig 2A and 2B). Additionally, as the oestrous progressed and follicles increased (SF → MF → LF), the concentrations of ITLN1 in FF from LW and MS pigs elevated. Comparing the two tested breeds of pigs, we observed that the mean of ITLN1 concentrations in FF on days 10–12, as well as days 14–16, of the oestrous was higher in LW pigs compared with MS pigs, whereas on days 2–3 of the oestrous, there were no significant differences in ITLN1 concentrations between LW and MS pigs (p < 0.05; Fig 2C). The precise values (F-statistic and p-value) for the main effects (*phase of oestrous*: 2–3; 10–12; 14–16 and *breed of pigs*: LW; MS) and the interactions of examined factors (phase*breed) are presented in S2 Table.

### Immunolocalization of ITLN1 in the ovarian follicles from LW pigs

**Fig 3:**
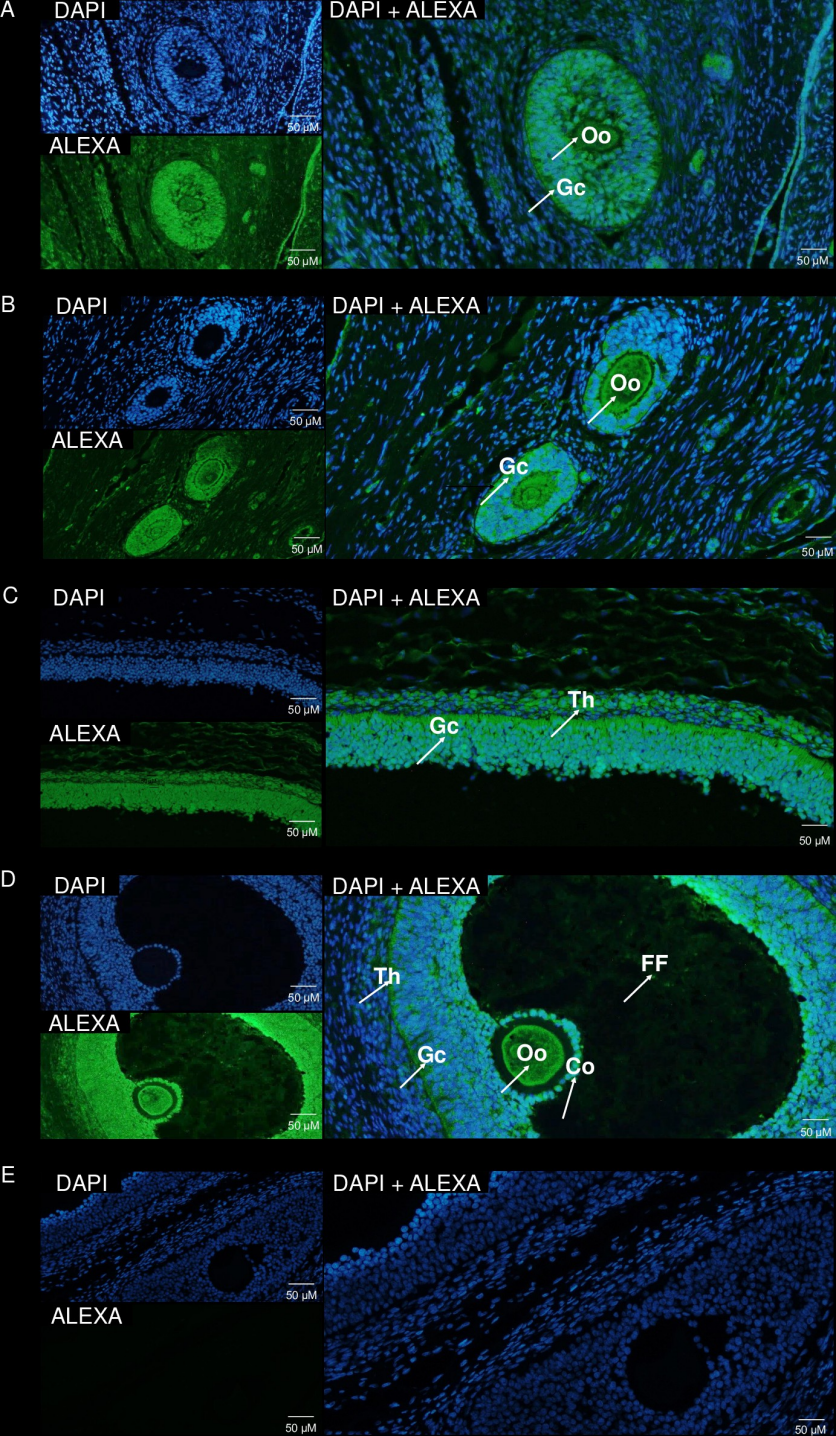
The localization of ITLN1 in the ovarian structures from LW pigs. The pictures show representative images of the immunolocalization of ITLN1 determined in the ovarian tissue slices (n = 3 per group) obtained on days 2–3, 10–12, and 14–16 of the oestrous cycle. Nuclear staining with DAPI is shown in pictures marked as DAPI, and localization of ITLN1 protein detected by immunocomplexes labeled with Alexa Fluor® 488 is shown in pictures marked as ALEXA, while the merged images are marked as DAPI + ALEXA. Arrows indicate ITLN1 presence. C, cumulus cells; FF, follicular fluid; Gc, granulosa cells; LW, Large White; ITLN1, intelectin-1; Th, theca interna cells; Oo, oocyte.

The immunolocalization of ITLN1 was confirmed in the cytoplasm as well as in the cell membrane of Gc, oocyte (Fig 3A–3D), and Th (Fig 3C and 3D) cells in antral follicles. Moreover, during ovarian follicles increasing, we noted ITLN1 expression in FF and cumulus cells (Fig 3D). No immunoreaction was observed for the negative controls (Fig 3E).

### *In vitro* effect of gonadotropins on ITLN1 level in the ovarian follicle cells of LW and MS pigs

**Fig 4:**
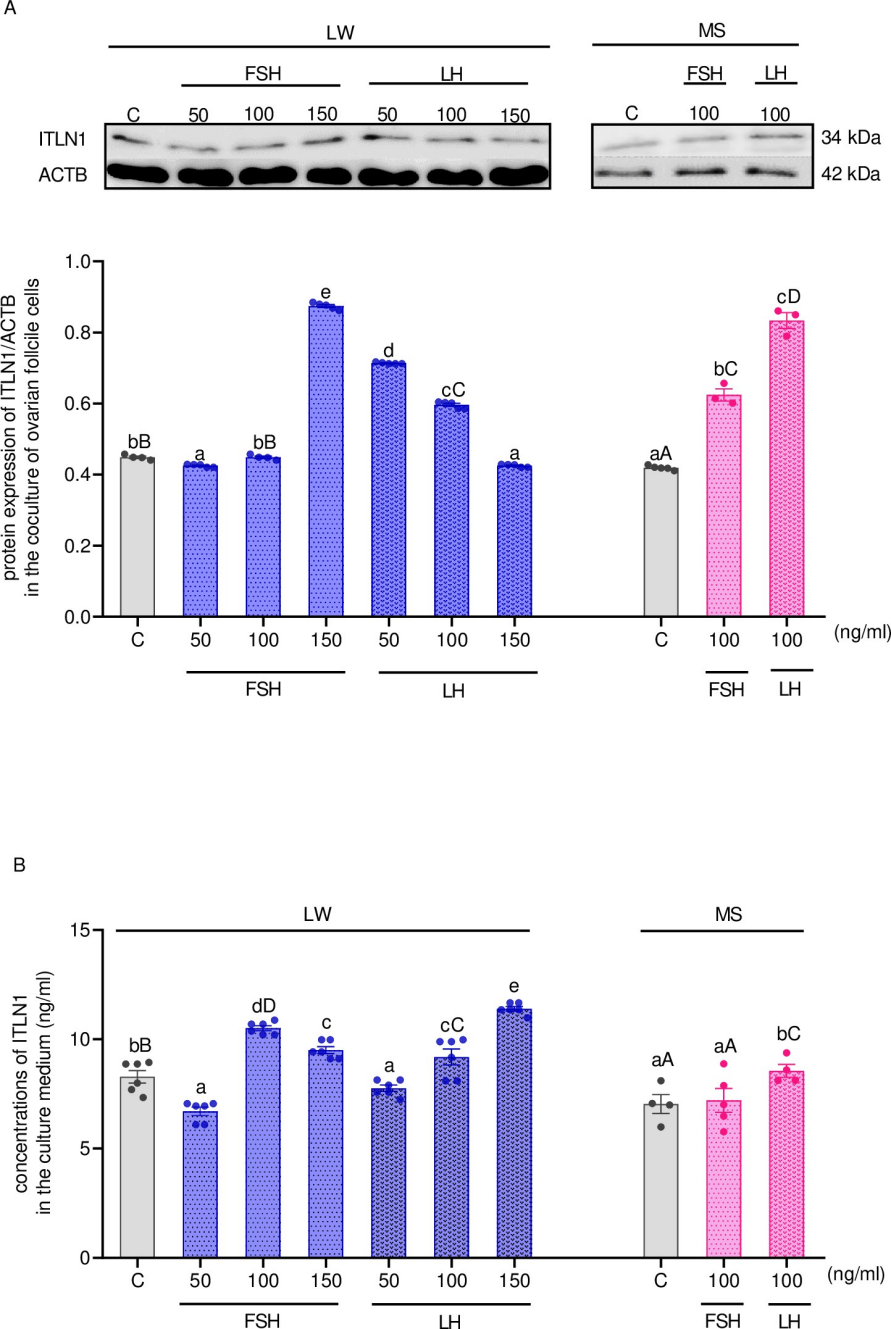
The effect of FSH and LH at doses of 50–150 ng/ml on ITLN1 protein expression (A) and its concentrations (B) in the culture medium of coculture of ovarian Gc and Th from LW and MS pigs on days 10–12 of the oestrous cycle. The protein expression was detected by western blot assay; each protein abundance was evaluated densitometrically and expressed as the ratio relative to ACTB abundance. ITLN1 concentration was determined by ELISA assay. Results were reported as the mean ± SEM of five independent determinations. The lower case letter denotes differences between hormones doses whereas an upper case letter denotes differences between types of pig breeds (p < 0.05; one-way ANOVA followed by Tukey post hoc test). ACTB, actin beta; ANOVA, analysis of variance; ELISA, enzyme-linked immunosorbent assay; FSH, follicle-stimulating hormone; LH, luteinizing hormone; LW, Large White; MS, Meishan; SEM, standard error of the mean; Tukey’s HSD, Tukey’s honest significant difference.

We next investigated the dose-dependent effect of gonadotropins on ITLN1 expression in the ovarian cells collected from MF. In LW pigs, FSH decreased ITLN1 protein expression at 50 ng/ml and increased at 150 ng/ml; on the other hand, no effect was noticed at a dose of 100 ng/ml. In MS pigs, FSH at the dose of 100 ng/ml elevated the protein expression of ITLN1. Additionally, in both breeds of pigs, LH had a stimulatory effect at 100 ng/ml. In LW pigs, we also noted that LH at the dose of 50 ng/ml increased ITLN1 protein expression, while inhibiting it at a dose of 150 ng/ml (Fig 4A, p < 0.05). Moreover, in LW pigs, FSH and LH at the doses of 100 and 150 ng/ml increased the concentrations of ITLN1 in the culture medium; also in MS pigs, only LH upregulated ITLN1 level in the culture medium. Interestingly, when comparing both breeds of pigs, the higher ITLN1 concentrations were observed in LW pigs compared with MS pigs after FSH and LH treatment at the dose of 100 ng/ml (Fig 4B, p < 0.05). The precise values (F-statistic and p-value) for the main effects and the interactions of examined factors are presented in S2 Table.

### *In vitro* effect of steroids on ITLN1 level in the ovarian follicle cells of LW and MS pigs

**Fig 5:**
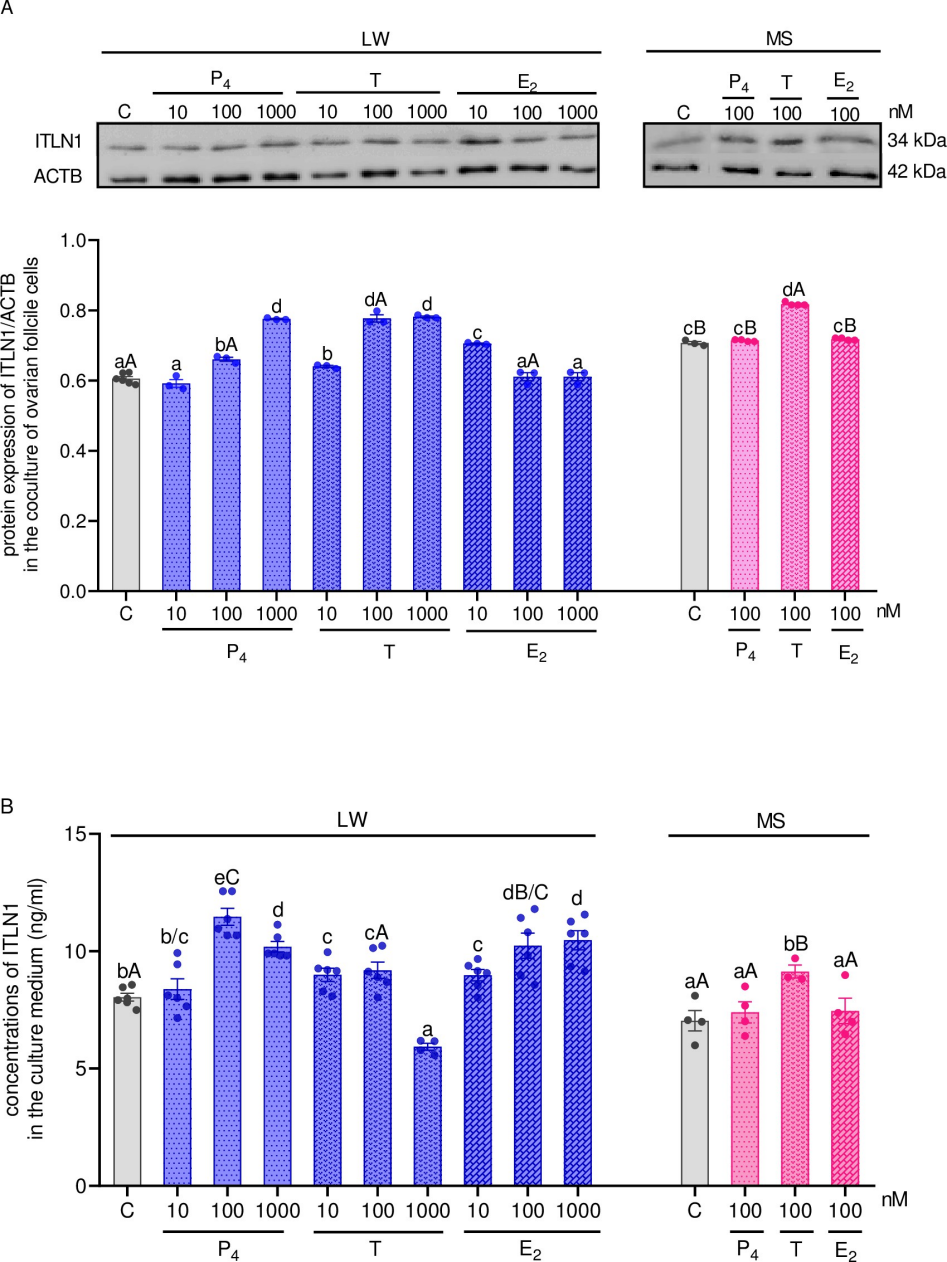
The effect of P_4_, T, and E_2_ at doses of 10–1000 nM on ITLN1 protein expression (A) and its concentrations (B) in the culture medium of coculture of ovarian Gc and Th from LW and MS pigs on days 10–12 of the oestrous cycle. The protein expression was detected by western blot assay; each protein abundance was evaluated densitometrically and expressed as the ratio relative to ACTB abundance. ITLN1 concentration was determined by ELISA assay. Results were reported as the mean ± SEM of five independent determinations. The lower case letter denotes differences between hormones doses whereas an upper case letter denotes differences between types of pig breeds (p < 0.05; one-way ANOVA followed by Tukey HSD test). ACTB, actin beta; ANOVA, analysis of variance; ELISA, enzyme-linked immunosorbent assay; E_2_, 17β-estradiol; LW, Large White; P_4_, progesterone; SEM, standard error of the mean; T, testosterone; Tukey’s HSD, Tukey’s honest significant difference.

In ovarian cells of LW pigs, P_4_ at the doses of 100 and 1000 nM, T at all studied doses, and E_2_ at the dose of 10 nM increased ITLN1 protein expression, whereas in MS pigs, only T (100 nM) stimulated ITLN1 protein level (Fig 5A, p < 0.05) in the ovarian follicular cells from MF. Additionally, we showed that ITLN1 concentrations in the culture medium from LW pigs were higher after stimulation with P_4_ at the doses of 100 and 1000 nM, as well as with E_2_ at the doses of 10–1000 nM. We also noted that T at a dose of 1000 nM significantly decreased ITLN1 concentrations compared to control group in LW pigs; however, the concentrations of ITLN1 in the culture medium of MS pigs were significantly enhanced only after T stimulation compared to control group (Fig 5B, p < 0.05).

### Involvement of ERK1/2 and PI3K signaling pathways in the regulatory action of gonadotropins on ITLN1 level in LW pigs

**Fig 6:**
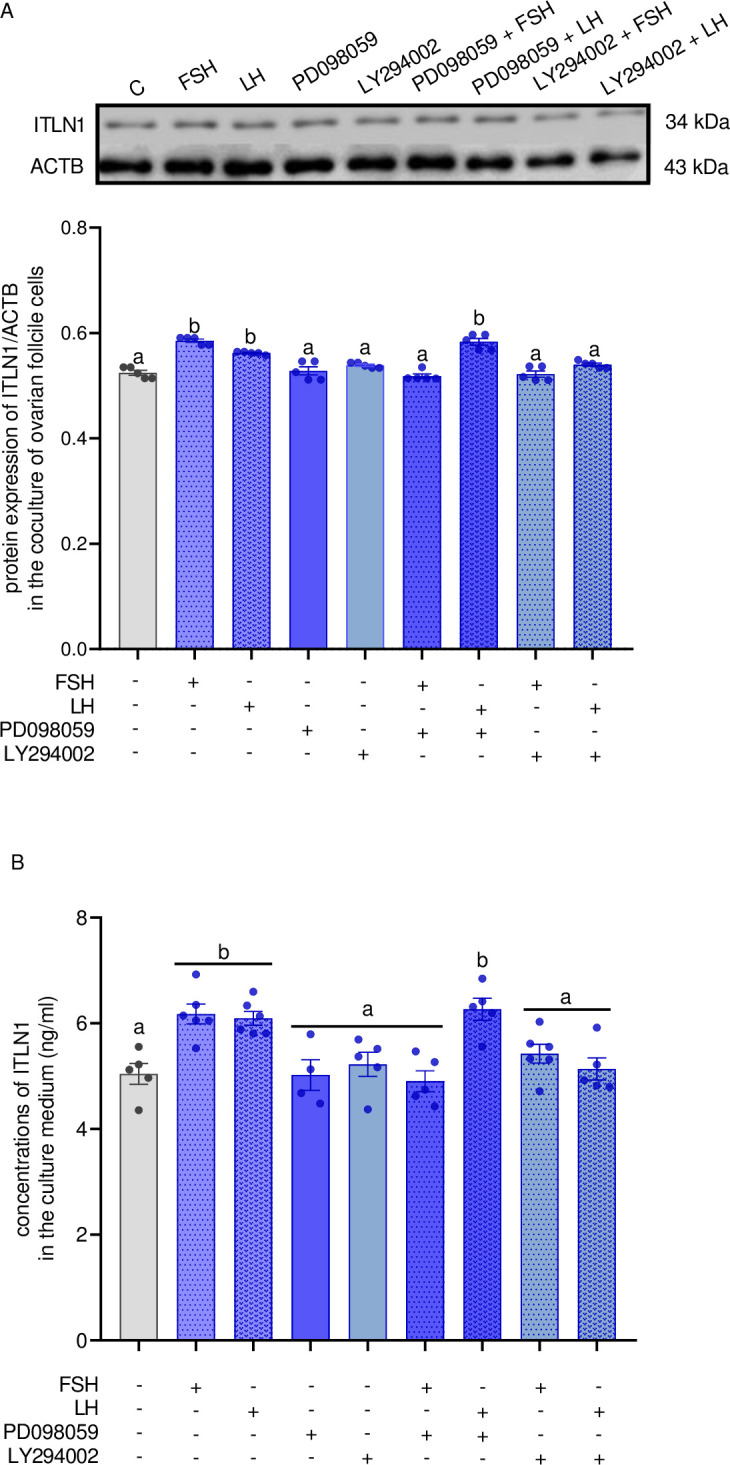
The effect of FSH and LH at a dose of 100 ng/ml on ITLN1 protein expression (A) and its concentrations (B) in medium of coculture of ovarian Gc and Th at days 10–12 of the oestrous cycle of LW pigs after using pharmacological inhibitors of ERK1/2 (PD098059, 50μM) and PI3K (LY294002, 10 μM). The protein expression was detected by western blot assay; each protein abundance was evaluated densitometrically and expressed as the ratio relative to ACTB abundance. ITLN1 concentration was determined by ELISA assay. Results were reported as the mean ± SEM of five independent determinations. Different letters denote statistically significant differences (p < 0.05; one-way ANOVA followed by Tukey’s HSD test). ACTB, actin beta; ANOVA, analysis of variance; ELISA, enzyme-linked immunosorbent assay; ERK1/2, extracellular signal-regulated kinase; FSH, follicle-stimulating hormone; LH, luteinizing hormone; LW, Large White; PI3K, phosphatidylinositol 3′-kinase; SEM, standard error of the mean; Tukey’s HSD, Tukey’s honest significant difference.

As shown in Fig 6A, ITLN1 protein expression was significantly higher in the ovarian cells treated with LH and PD098059. No significant differences were observed in ITLN1 protein levels after incubation with LY294004 in combination with FSH and LH (p < 0.05). Such results were confirmed by ITLN1 concentrations in the culture medium; we noted that PD098059 with LH increased secretion of ITLN1 (Fig 6B, p < 0.05) compared to cells treated only with PD098059.

### Involvement of ERK1/2 and PI3K signaling pathways in the regulatory action of steroids on ITLN1 level in LW pigs

**Fig 7:**
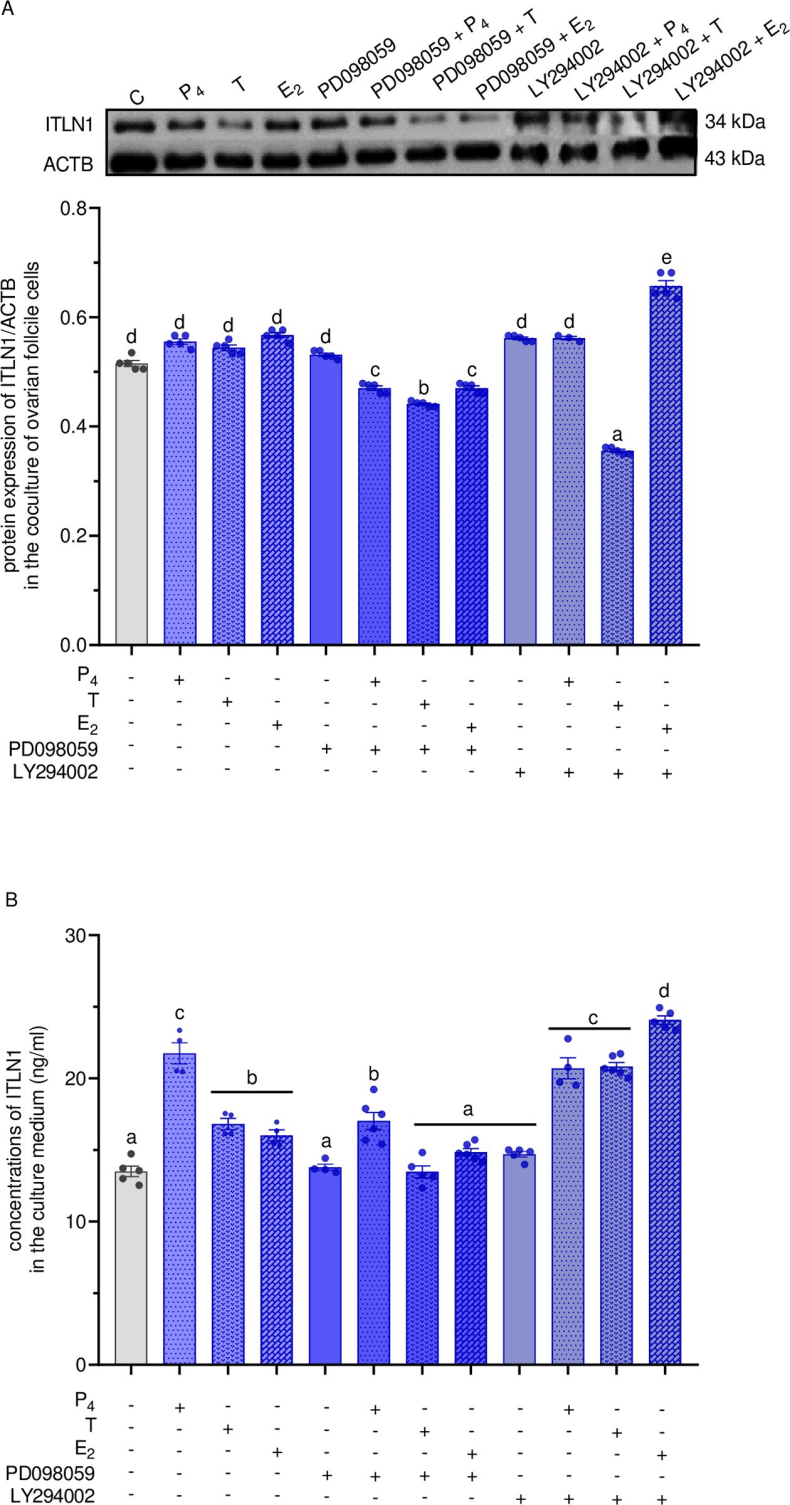
The effect of P_4_, T, and E_2_ at a dose of 100 nM on ITLN1 protein expression (A) and concentrations (B) in medium of coculture of ovarian Gc and Th at days 10–12 of the oestrous cycle of LW pigs after using pharmacological inhibitors of ERK1/2 (PD098059, 50μM) and PI3K (LY294002, 10μM). The protein expression was detected by western blot assay; each protein abundance was evaluated densitometrically and expressed as the ratio relative to ACTB abundance. ITLN1 concentration was determined by ELISA assay. Results were reported as the mean ± SEM of five independent determinations. Different letters denote statistically significant differences (p < 0.05; one-way ANOVA followed by Tukey’s HSD test). ACTB, actin beta; ANOVA, analysis of variance; E_2_, 17β-estradiol; ELISA, enzyme-linked immunosorbent assay; ERK1/2, extracellular signal-regulated kinase; FSH, follicle-stimulating hormone; LH, luteinizing hormone; LW, Large White; MS, Meishan; P_4_, progesterone; PI3K, phosphatidylinositol 3′-kinase; SEM, standard error of the mean; T, testosterone; Tukey’s HSD, Tukey’s honest significant difference.

We noted that the protein contents of ITLN1 were elevated only in cells treated with E_2_ and LY294002. The opposite effect was observed in cells treated with PD098059 and all studied steroid hormones, as well as LY294002 in combination with T (Fig 7A, p < 0.05). On the other hand, ITLN1 secretion to the culture medium increased after treatment with PD098059 and P_4_ compared to treatment only with PD098059, as well as with LY294002 in combination with P_4_, T, and E_2_ compared to cells treated only with LY294002 (Fig 7B, p < 0.05).

## Discussion

ITLN1 is a novel adipokine associated with the maintenance of body homeostasis, whose role in the reproduction is still unknown. In our study, we confirmed the hypothesis that ITLN1 expression (gene and protein), as well as its concentrations in FF, in pigs depends on fat quantification. For the first time, we noted that obese MS pigs have the lower ITLN1 levels in the ovary compared to normal weight LW pigs; a similar phenomenon has been shown by Barbe *et al*. [13] in the peri renal adipose tissue and serum. Also, the differences in adipokine ovarian expression are well described in the literature; Nakajima *et al*. [25] showed lowered adiponectin gene expression in MS pigs compared to normal weigh Landrace pigs and Kurowska *et al*. [46] noted the opposite pattern of vaspin expression in LW and MS pigs (LW > MS). The explanation of observed differences is that generally obese humans and rodents are characterized by an excessive mass of adipose tissue accompanied by an increase in circulating glucose, insulin, and triglyceride levels [49], which may interfere with adipokine content. For example, Tan *et al*. [16] showed that in the omental adipose tissue explants, insulin dose-dependently decreased the gene and protein expression of ITLN1, as well as its secretion into conditioned medium. In agreement with our observation, MS pigs have the higher homeostasis model assessment-estimated insulin resistance (HOMA-IR) score and triglyceride amount [33] compared to LW pigs, which negatively correlate with ITLN1 plasma concentrations [50]. Negative correlations between the ITLN1 expression and the percentage of body adipose tissue in obese people were noted [50], which is in good agreement with the results obtained in this study.

We noted that ITLN1 levels in the ovarian follicles and its concentrations in FF depends on the oestrous cycle phase. We observed that ITLN1 levels increase with the progression of the oestrous cycle in both breeds. The differences between gene and protein expression noted on days 2–3 of the oestrous are probably dependent on the posttranscriptional regulation: 5′-end capping to generate a 7-methylguanosine cap, splicing out of introns, and 3′-end cleavage/polyadenylation [51] because, as Schwanhäusser *et al*. [52] suggested, dissimilarity in gene expression explained approximately 40% of the variation in protein levels. The observed pattern of ITLN1 expression during the oestrous in pigs and higher levels in the LW pigs compared to MS pigs may be clarified by the fact that on day 20 of the oestrous, LW gilts have a higher number of Gc, FF volume, and Th mass [53] which, as we showed, express ITLN1. Additionally, to confirm our results, previous papers have documented that MS and LW gilts have elevated aromatase activity in Gc and Th [53] and plasma levels of E_2_ [54] during both the early and late follicular phases, which negatively correlates with ITLN1 concentrations [16]. In our study, we also noted that ITLN1 levels in FF of both tested breeds of pigs increased during the oestrous cycle; on the other hand, ITLN1 plasma concentrations in women was stable during the menstrual cycle [55]. Such differences may be explained by the fact that FF that fills the antrum of the growing follicle is derived from both the blood constituents that cross the blood-follicle barrier and products of the follicle structures [56]. We showed immunolocalization of ITLN1 in the cytoplasm and cell membrane in the ovarian follicles collected from a the different phases of the oestrous cycle in LW pigs. ITLN1 was present mainly in Gc, Th, oocyte, cumulus cells, and FF; the same pattern of ITLN1 has been described in the compartments of human ovaries by Cloix *et al*. [10]. Interestingly, we noted strong expression of ITLN1 in the zona pellucida of ovarian follicles before ovulation, suggesting the role of ITLN1 in the oocyte maturation.

Based on results indicating that the expression of ITLN1 was changing during the oestrous cycle, in the second aim, we derive novel observations about the regulation of ITLN1 in the ovarian follicle cells by gonadotropins and steroids, which participates in the oestrous cycle progression and regulation of ovarian follicle growth, ovulation and corpus luteum formation [46, 57]. In our study, we observed that gonadotropins and steroid hormones increased ITLN1 levels in LW pigs in a dose-dependent manner. Previous studies have reported that gonadotropins and steroids are involved in the regulation levels of another adipokine, vaspin; similarly, Kurowska *et al*. [46] documented that FSH, LH, P_4_, T, and E_2_ increased vaspin levels in the ovarian follicle of LW pigs. These findings are consistent with the results obtained by us for LW pigs and suggest that ITLN1 levels during the oestrous may be regulated by hormones involved in its progression. Interestingly, Cloix *et al*. [10] documented that INTL1 expression was inhibited after 48 h of treatment with FSH at the dose of 10 nM in non-obese human Gc and the KGN cell line. In our study, after 48 h, we also observed the reduction of ITLN1 expression and its secretion to culture medium by ovarian cells in LW pigs after FSH treatment at the dose of 50 ng/ml (1.5 nM). At this point, it is worth adding that the higher dose of FSH used by Cloix *et al*. [10] may be connected with lowered gene expression of the FSH receptor in KGN cell line [58] than in the primary culture of ovarian follicle cells.

We also noted a different pattern in the regulation of ITLN1 levels in LW and MS pigs because we observed only the stimulatory effect of LH and T on ITLN1 levels in MS pigs. We suggest that such differences may be associated with the alternative endocrine relationship between LW and MS pigs. For example, Tilton *et al*. [59] noted increased levels of gonadotropins in normal weight pigs compared to fat MS pigs, which may be explained by differential binding of gonadotropin to FSH and LH receptors. We suggest that the stimulatory effect only by one gonadotropin (LH) in MS pigs on ITLN1 levels may be associated with elevated levels of plasma cortisol in MS pigs compared to lean breeds of pigs [54], which can intensify LH response in Gc of rats [60] and may also occur in MS pigs because they demonstrated earlier estrus and shorter intervals between LH surge compared to LW pigs [53]. Moreover, MS pigs have higher inhibin levels which reduce the secretion of FSH from the pituitary gland and the expression of its receptor and may give an explanation for FSH having no effect on ITLN1 levels [54]. At this point, it is worth adding that no impact of E_2_ on ITLN1 levels in MS pigs may be connected with a higher concentrations of E_2_ in FF and plasma compared to LW pigs [61]. We propose that higher levels of E_2_ in both plasma and FF of MS pigs (S1 Fig) may be associated with E_2_ receptor desensitisation linked with the decreased responsiveness of E_2_ that occurs after repeated or chronic exposure. Moreover, we assess the stimulatory effect of only one steroid, T, on ITLN1 levels in MS pigs. Previous studies indicated only higher plasma levels of T in MS boars compared to normal weight Duroc pigs and white composite breed, and the increased free T level is accompanied by decreased plasma levels of ITLN1 levels in obese women [62]. Based on this interesting findings in MS pigs, we also suggest that different ITLN1 regulation between these two types of breeds may be linked with hypothesize that LW and MS pigs may exhibit a different pattern in the expression and sensitivity of LH, FSH, P_4_, T, and E_2_ receptors in the ovarian follicle cells, which we plan to study in our future experiments.

Finally, we investigated the involvement of ERK1/2 and PI3K in the regulation of ITLN1 expression in response to gonadotropins and steroids in LW pigs. We focused on these kinases because signalling pathways, such as Janus kinase, ERK1/2, PI3K, adenosine 5′-monophosphate-activated protein kinase, and nuclear factor-κB, were activated in vaspin regulation in the cultured ovarian cells in LW pigs [46]. Moreover, available literature data suggest that ITLN1 may suppress tumour necrosis-α-induced expression of endothelial adhesion molecules and cyclooxygenase-2 in the primary human umbilical vein endothelial cells via ERK1/2 [63] and stimulate the human osteoblast proliferation through PI3K [64]. In our study, we observed that specific inhibitors of ERK1/2 and PI3K abolish the stimulating effect of both gonadotropins and steroids on ITLN1 levels, suggesting that the expression of this adipokine in the ovarian cells can be mediated via these kinases. It is worth adding that in this part, some obtained results showed the incoherence between ITLN1 protein expression and corresponding ITLN1 secretion to the culture medium; however, as Praznik *et al*. [65] suggested, not all proteins that enter the endoplasmic reticulum (ER) are secreted. Some proteins exert their function inside ER and need to be retained there or recycled between ER and post-ER compartments, and the processes of transcription and translation can require several hours before the protein is secreted to the functionally relevant concentrations.

In summary, our data showed that: *i)* ITLN1 expression was significantly higher in the ovarian follicles and FF from LW pigs compared to MS pigs; levels of ITLN1 in the ovarian follicles and its concentrations in FF increased in LW and MS pigs during the oestrous cycle; and ITLN1 signal is present in Gc, Th, and oocyte; *ii)* ITLN1 levels were increased by FSH, LH, P_4_, E_2_, and T in LW pigs, while in MS pigs, we observed only the stimulatory effect of LH and T; and *iii)* ERK1/2 and PI3K are associated in the regulation of ITLN1 levels in the ovarian cells. The latest literature data noted that ITLN1 is associated with the pathology of ovaries, like ovarian cancer and PCOS linked with obesity; interestingly the ovarian cancer cell line treated with ITLN1 metastasis was inhibited [66]. For this reason, further studies are necessary to understand the role of ITLN1 as a new regulator in the ovarian physiology (for example, steroid secretion, cell proliferation, apoptosis, and oocyte maturation), thus providing new insights about their function in the reproduction of animals with different metabolic status.

## Supporting information

S1 Fig Orginal blots. Abbreviation: LW, Large White; MS, Meishan, C, control; FSH, follicle stimulating hormone; LH, luteinizing hormone; P_4_, progesterone; T, testosterone; E_2_, 17β-estradiol.
(DOCX)

S1 Table Sources of reagents used in the study. Abbreviation: BSA, bovine serum albumin; HRP, horseradish peroxidase; E_2_, 17β-estradiol; FBS, fetal bovine serum; FSH, follicle stimulating hormone; LH, luteinizing hormone; P_4_, progesterone; PBS, phosphate buffered saline; PVDF, polyvinylidene fluoride; T, testosterone.
(DOCX)

S2 Table The effect of main factors and their interaction on mRNA and protein expression of ITLN1 in the ovarian follicles as well as its concentration in FF. Abbreviation: FF, follicular fluid; ITLN1, intelectin; phase, 2-3/10-12/14-16 of the oestrous cycle; breed, Large White/Meishan;*, interaction.
(DOCX)

S3 Table Concentration of P4, E2 in plasma and E2 in follicular fluid of Large White and Meishan pigs during the oestrous cycle. Abbreviation: E_2_, 17β-estradiol; P_4_, progesterone. The hormone concentrations were evaluated using ELISA. Results are presented as at least eight independent replicates as means ± SEM for each group. Statistical significance is indicated by different letters (p < 0.05).
(DOCX)

S1 Graphical abstract E_2_, 17β-estradiol; ERK1/2, extracellular signal-regulated kinase; FF, follicular fluid; FSH, follicle-stimulating hormone; ITLN1, intelectin 1; LH, luteinizing hormone; LW, Large White; MS, Meishan; P_4_, progesterone; PI3K, phosphatidylinositol 3′-kinase; T, testosterone. (PDF)
