# Supplementary material for: Omentin expression in the ovarian follicles of Large White and Meishan sows during the oestrous cycle and in vitro effect of gonadotropins and steroids on its level: Role of ERK1/2 and PI3K signaling pathways
Source: PLoS One. 2024 Feb 26;19(2):e0297875. doi: 10.1371/journal.pone.0297875 (PMC10896505; doi:10.1371/journal.pone.0297875)
Supplement: S1 Fig — Abbreviation: LW, Large White; MS, Meishan, C, control; FSH, follicle stimulating hormone; LH, luteinizing hormone; P4, progesterone; T, testosterone; E2, 17β-estradiol. (DOCX) [file pone.0297875.s001.docx]

**ORGINAL BLOTS**

**FIGURE 2A**

Anti-INTELECTIN antibody diluted at 1:500.


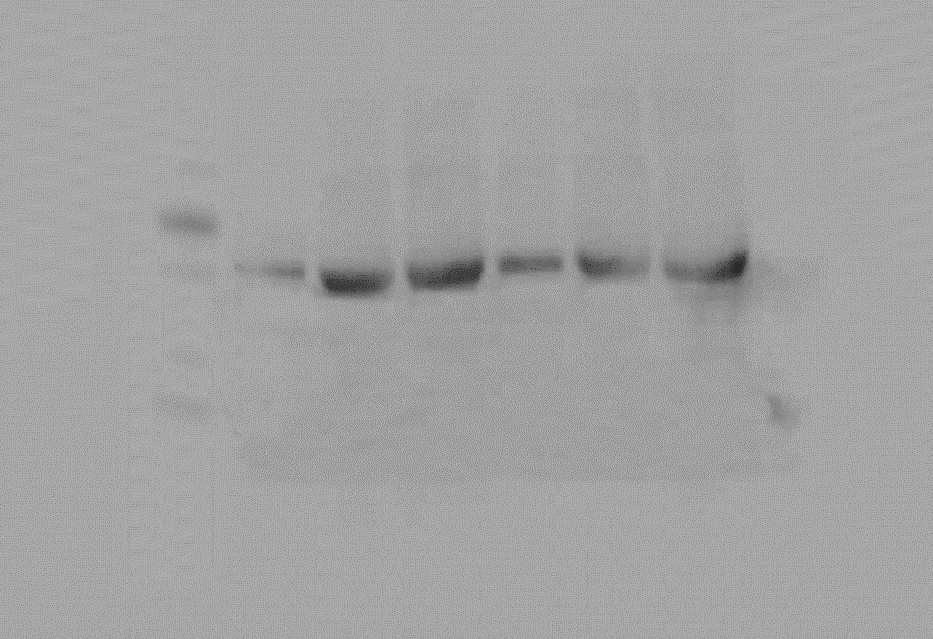


days of oestrous cycle

**M**

**MS LW MS LW MS LW**

protein ladder

55 kDa

14-16

10-12

2-3

**days of oestrous cycle**

**14-16**

**2-3**

INTELECTIN, 34 kDa

MS LW MS LW MS LW

Anti-β-ACTIN antibody diluted at 1:1000.


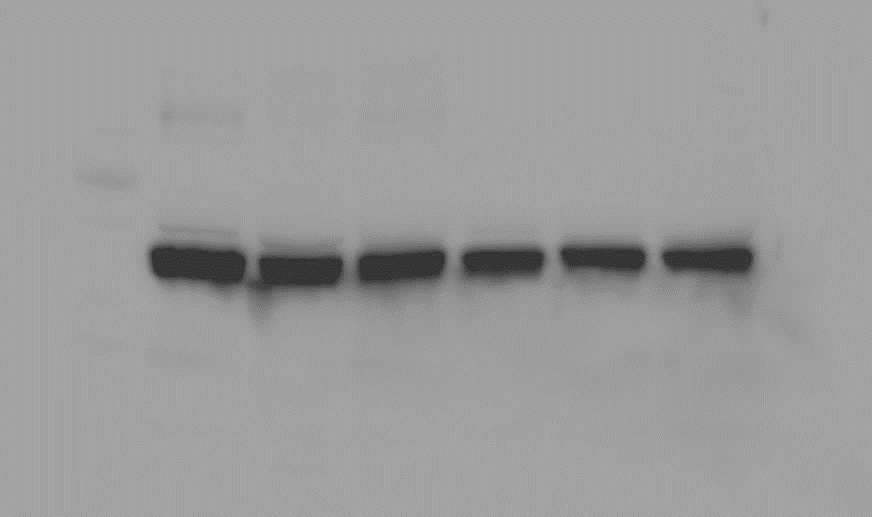


days of oestrous cycle cycle

β-ACTIN, 42 kDa

protein ladder

55 kDa

14-16

10-12

2-3

MS LW MS LW MS LW

*Abbreviations: LW- Large White, MS- Meishan.*

**FIGURE 4A**

Anti-INTELECTIN antibody diluted at 1:500.


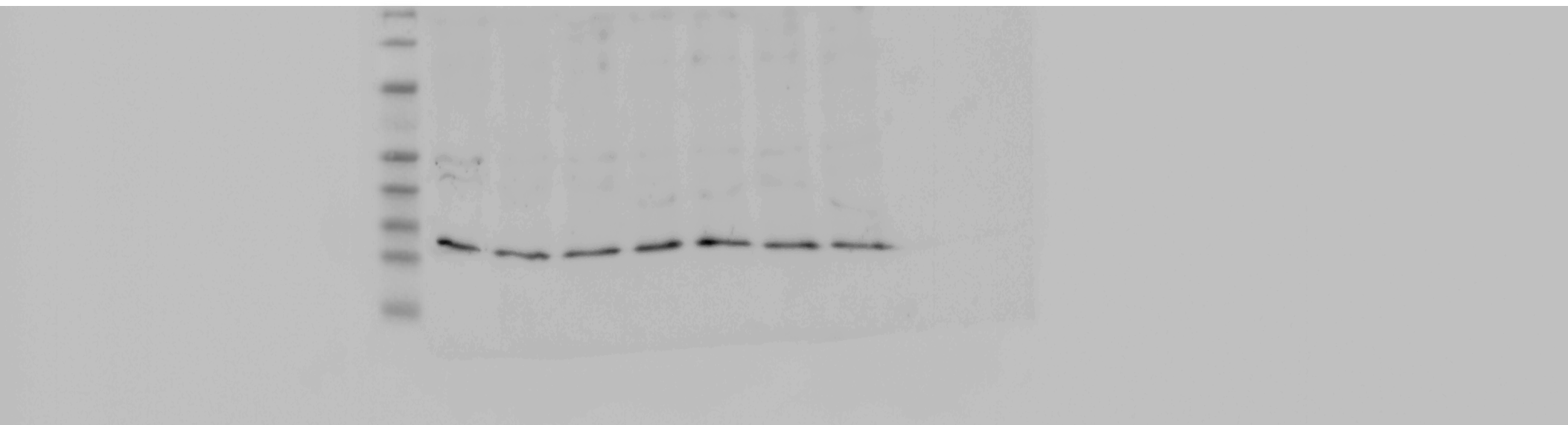


Large White

protein lader 55 kDa

FSH

LH

INTELECTIN, 34 kDa

C 50 100 150 50 100 150 ng/ml

Anti-β-ACTIN antibody diluted at 1:1000.


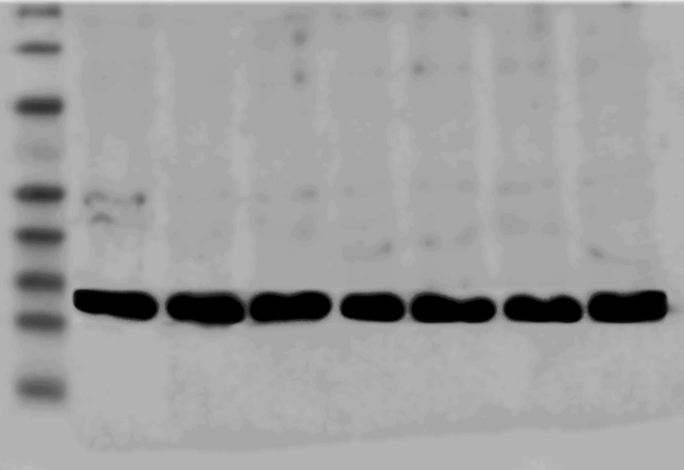


Large White

C 50 100 150 50 100 150 ng/ml

protein lader 55 kDa

LH

FSH

β-ACTIN, 42 kDa

*Abbreviations: C-control, FSH- follicle stimulating hormone, LH- luteinizing hormone.*

Anti-INTECETIN antibody diluted at 1:500.


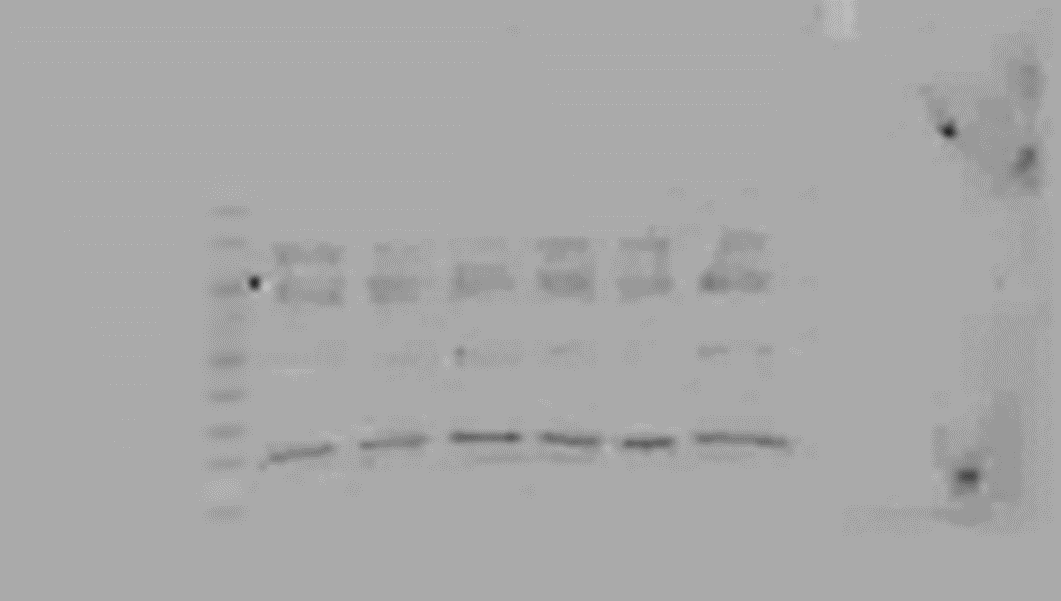


LH

FSH

protein lader 55 kDa

C 100 100 ng/ml

Meishan

INTELECTIN, 34 kDa

Anti-β-ACTIN antibody diluted at 1:1000.


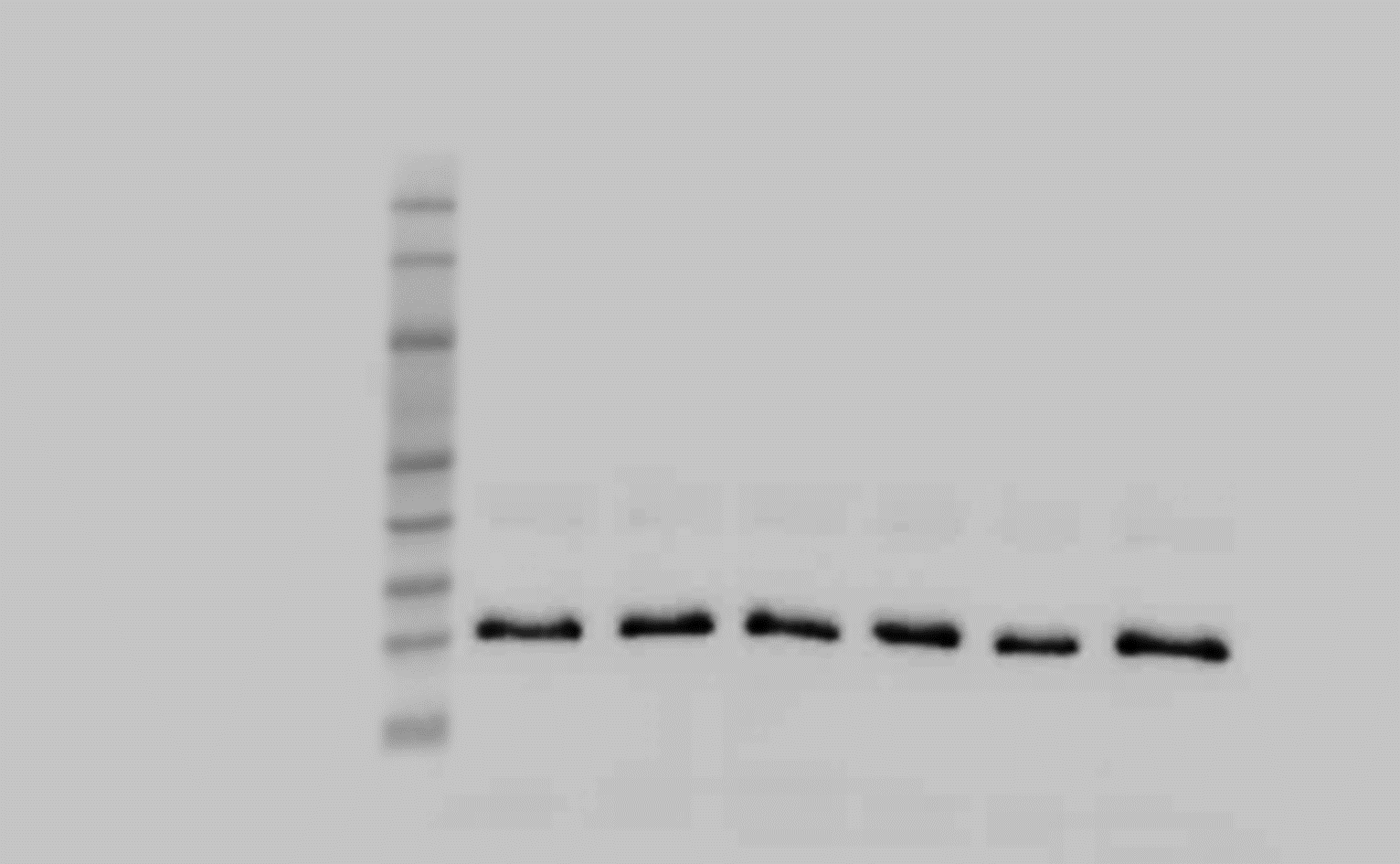


protein lader 55 kDa

C 100 100 ng/ml

Meishan

LH

FSH

β-ACTIN, 42 kDa

*Abbreviations: C-control, FSH- follicle stimulating hormone, LH- luteinizing hormone.*

**FIGURE 5A**

Anti-INTELECTIN antibody diluted at 1:500.


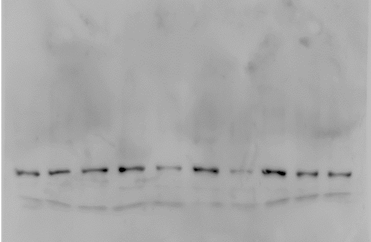


Large White

P_4_  T E_2_

INTELECTIN, 34 kDa

C 10 100 1000 10 100 1000 10 100 1000 nM

Anti-β-ACTIN antibody diluted at 1:1000.


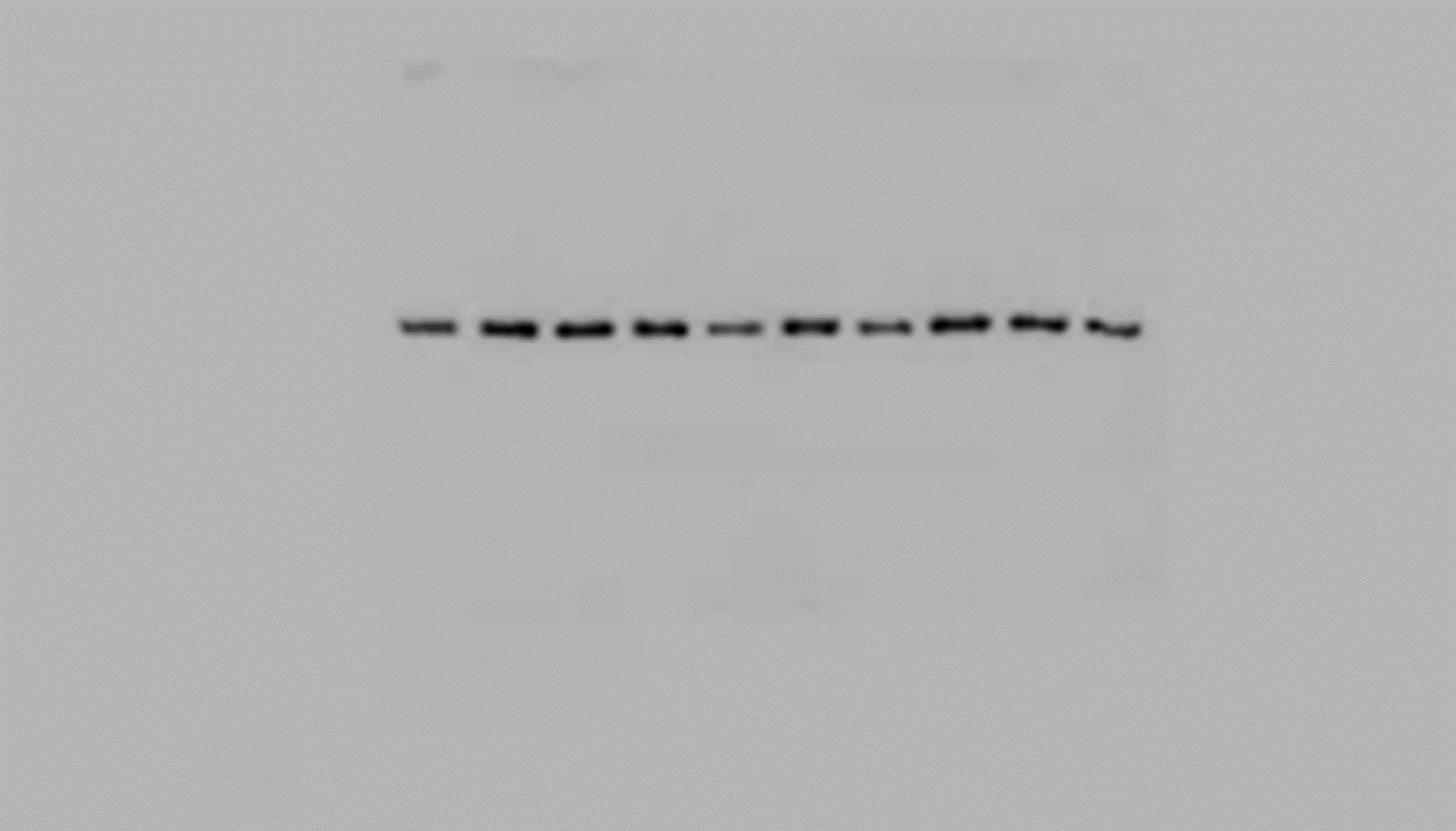


Large White

P_4_  T E_2_

β-ACTIN, 42 kDa

C 10 100 1000 10 100 1000 10 100 1000 nM

*Abbreviations: C-control, P_4_- progesterone, T- testosterone, E_2_- 17β*-*estradiol.*

Anti-INTELECTIN antibody diluted at 1:500.


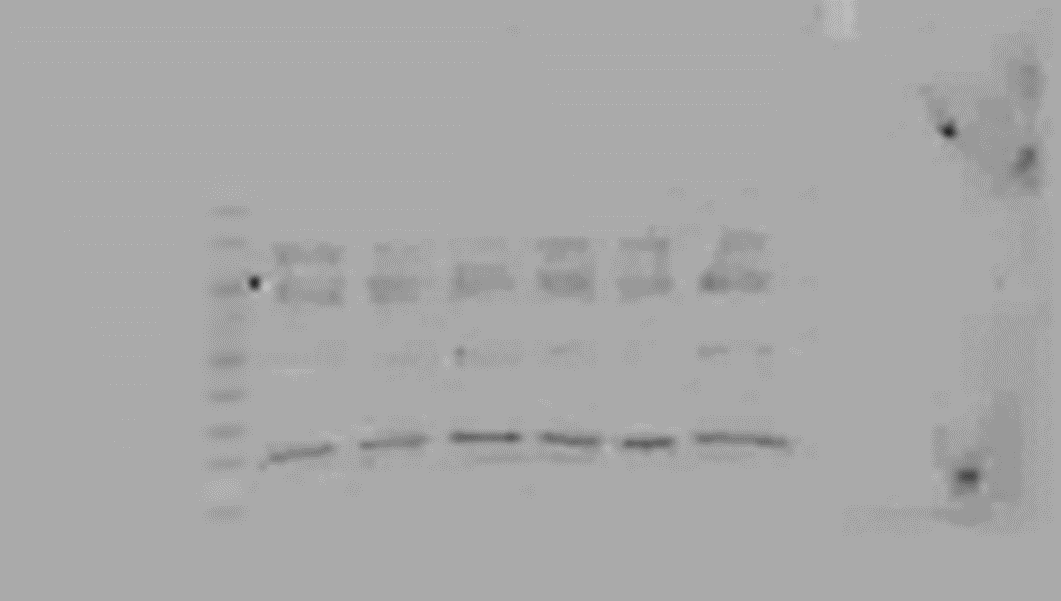


100 100 100 nM

protein lader 55 kDa

C

T

E_2_

P_4_

Meishan

INTELECTIN, 34 kDa

Anti-β-ACTIN antibody diluted at 1:1000.


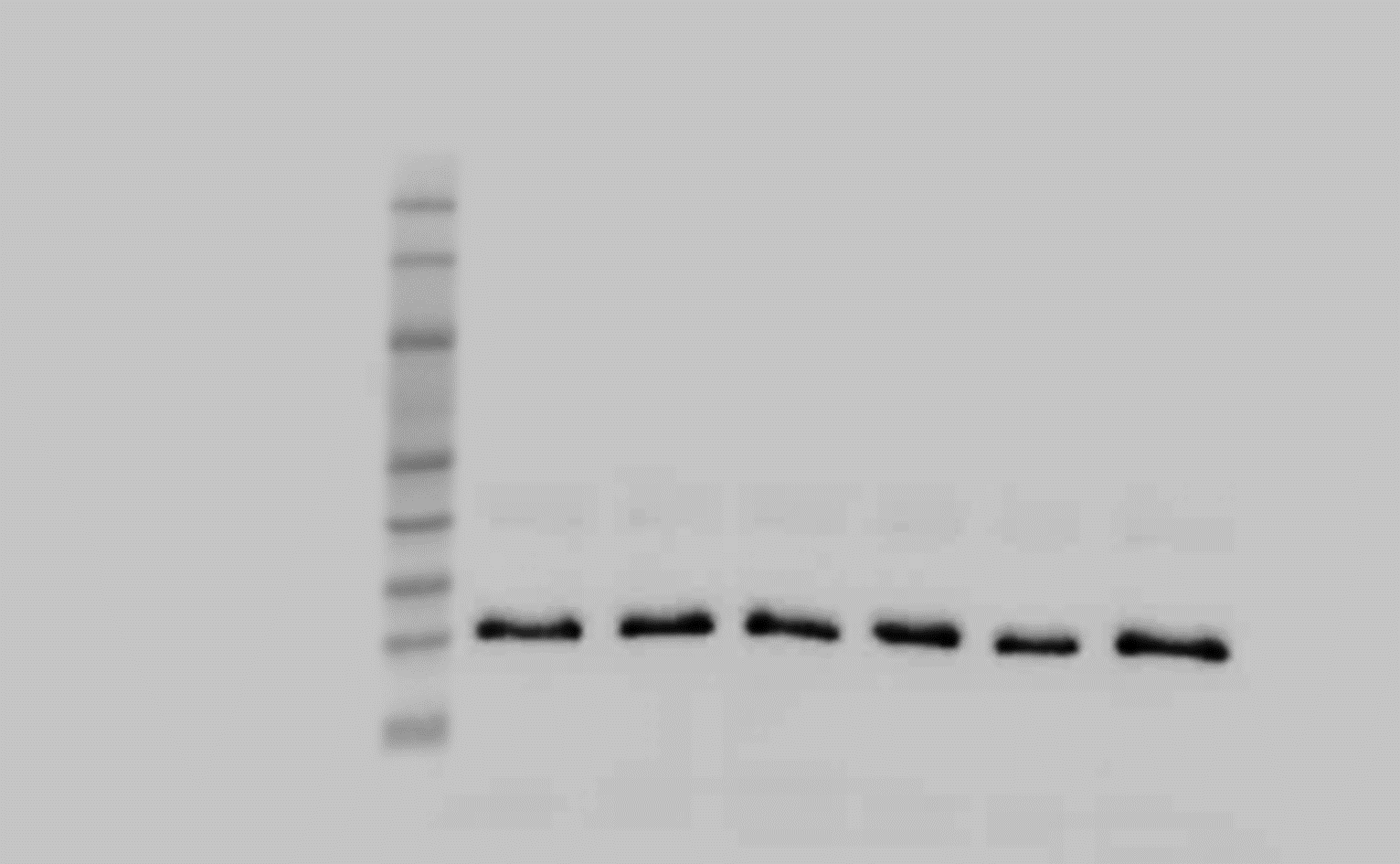


protein lader 55 kDa

C

100 100 100 nM

Meishan

β-ACTIN, 42 kDa

E_2_

T

P_4_

*Abbreviations: C-control, P_4_- progesterone, T- testosterone, E_2_- 17β*-*estradiol.*

**FIGURE 6A**

Anti-INTELECTIN antibody diluted at 1:500.


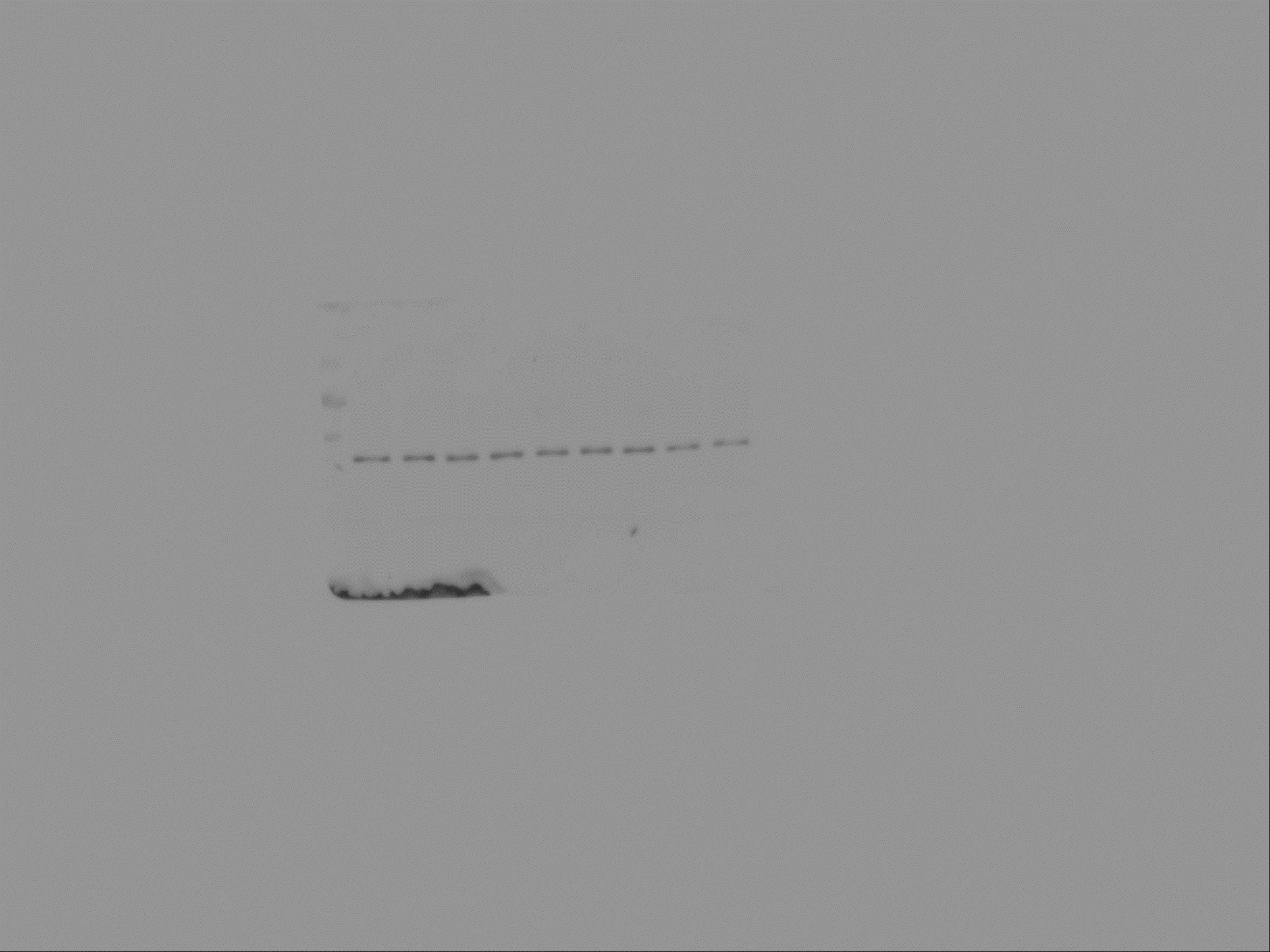


LY294002+LH

PD 098059+LH

PD 098059+FSH

LY294002+FSH

+FSH

protein lader 55 kDa

PD 098059

INTELECTIN, 34 kDa

LY294002

LH

FSH

C

Anti-β-ACTIN antibody diluted at 1:1000.


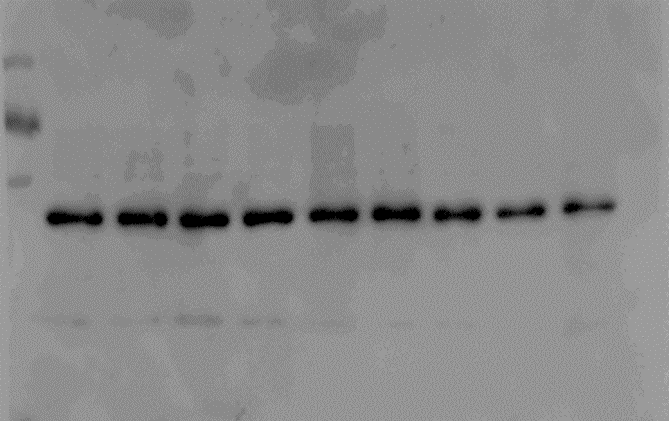


PD 098059+FSH

PD 098059+LH

LY294002+FSH

+FSH

LY294002+LH

protein lader 55 kDa

PD 098059

LY294002

β-ACTIN, 42 kDa

C

FSH

LH

*Abbreviations: C-control, FSH- follicle stimulating hormone, LH- luteinizing hormone.*

**FIGURE 7A**

Anti-INTELECTIN antibody diluted at 1:500.


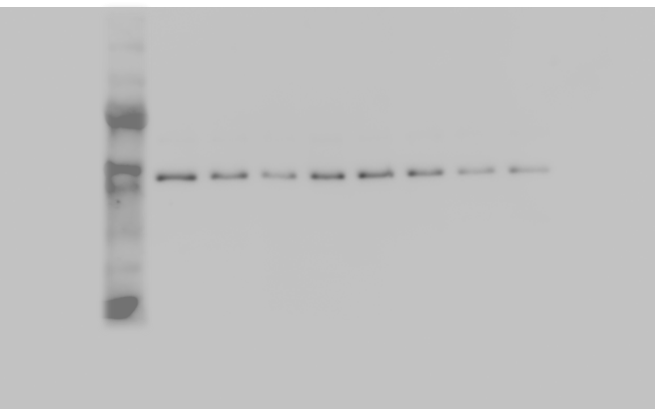


protein lader 55 kDa

E_2_

T

P_4_

C

PD 098059 +E_2_

PD 098059+P_4_

PD 098059+T

PD 098059

INTELECTIN, 34 kDa

Anti-β-ACTIN antibody diluted at 1:1000.


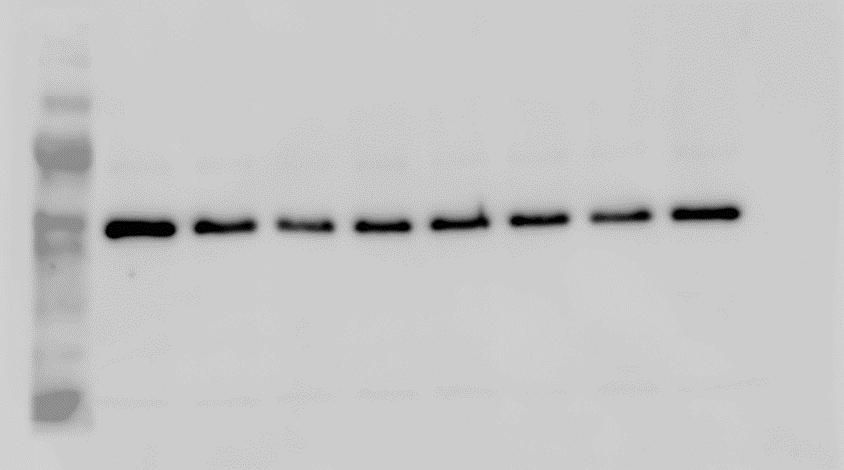


PD 098059+P_4_

PD 098059 +E_2_

PD 098059+T

PD 098059

protein lader 55 kDa

C

β-ACTIN, 42 kDa

E_2_

T

P_4_

*Abbreviations: C-control, P_4_- progesterone, T- testosterone, E_2_- 17β-estradiol.*

Anti-INTELECTIN antibody diluted at 1:500.


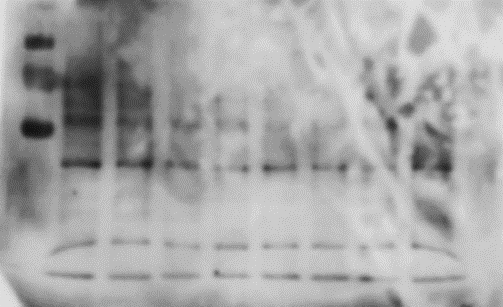


LY29400+E_2_

LY29400+T

LY29400+P_4_

protein lader 55 kDa

LY294002

INTELECTIN, 34 kDa

C

P_4_

T

E_2_

Anti-β-ACTIN antibody diluted at 1:1000.

*
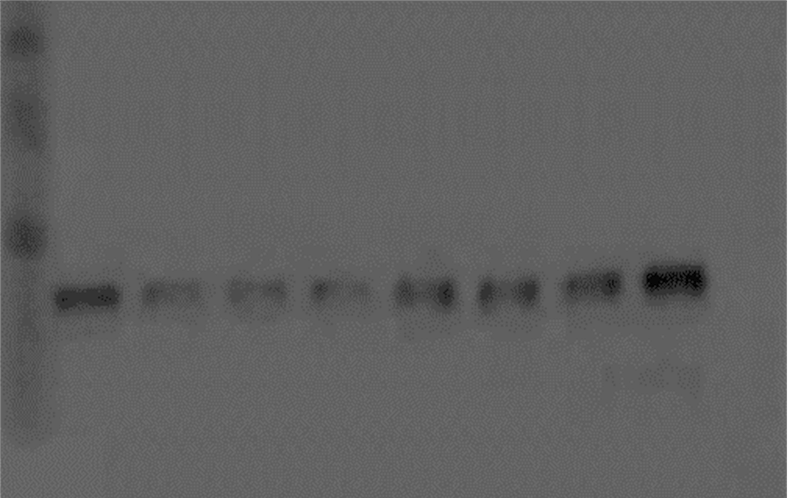
*

protein lader 55 kDa

β-ACTIN, 42 kDa

LY29400+E_2_

LY29400+T

LY29400+P_4_

LY294002

C

E_2_

T

P_4_

*Abbreviations: C-control, P_4_- progesterone, T- testosterone, E_2_-17β- estradiol.*
