# Supplementary material for: Omentin expression in the ovarian follicles of Large White and Meishan sows during the oestrous cycle and in vitro effect of gonadotropins and steroids on its level: Role of ERK1/2 and PI3K signaling pathways
Source: PLoS One. 2024 Feb 26;19(2):e0297875. doi: 10.1371/journal.pone.0297875 (PMC10896505; doi:10.1371/journal.pone.0297875)
Supplement: S1 Table — Abbreviation: BSA, bovine serum albumin; HRP, horseradish peroxidase; E2, 17β-estradiol; FBS, fetal bovine serum; FSH, follicle stimulating hormone; LH, luteinizing hormone; P4, progesterone; PBS, phosphate buffered saline; PVDF, polyvinylidene fluoride; T, testosterone. (DOCX) [file pone.0297875.s002.docx]

**Supplementary Table 1.** Sources of reagents used in the study.

| **REAGNET** | **CATALOGUE NUMBER** | **SUPPLIER** |
| --- | --- | --- |
| Bovine serum albumin | ALB001.500 | BioShop, Canada |
| Chemiluminescent HRP substrate reagent | WBKLS0500 | Sigma-Aldrich, United States |
| 17β-estradiol | E2257 | Sigma-Aldrich, United States |
| Fetal bovine serum | S181H-500 | Biowest, France |
| Follicle stimulating hormone | F4021 | Sigma-Aldrich, United States |
| iQ SYBR Green Supermix | 1708885 | Bio-Rad, United States |
| Laemmli buffer | 38733 | Sigma-Aldrich, United States |
| Luteinizing hormone | L5259 | Sigma-Aldrich, United States |
| LY29400 | 9901 | Cell Signaling Technology, United States |
| M199 medium | M4530 | Sigma-Aldrich, United States |
| Progesterone | P0130 | Sigma-Aldrich, United States |
| PBS | 14040117 | ThermoFisher, United States |
| PD098059 | 1213 | Tocris Bioscience, United Kingdom |
| PVDF membranes | IPVH00010 | Sigma Aldrich, United States |
| QIAzol Lysis Reagent | 79306 | Qiagen, Germany |
| Testosterone | 86500 | Sigma-Aldrich, United States |
| Trypan blue | 15250061 | ThemoFisher, United States |
| Trypsin | 15400054 | ThermoFisher, United States |

Abbreviation: HRP, horseradish peroxidase; PBS, phosphate buffered saline; PVDF, polyvinylidene fluoride;
