# Supplementary material for: Omentin expression in the ovarian follicles of Large White and Meishan sows during the oestrous cycle and in vitro effect of gonadotropins and steroids on its level: Role of ERK1/2 and PI3K signaling pathways
Source: PLoS One. 2024 Feb 26;19(2):e0297875. doi: 10.1371/journal.pone.0297875 (PMC10896505; doi:10.1371/journal.pone.0297875)
Supplement: S2 Table — Abbreviation: FF, follicular fluid; ITLN1, intelectin; phase, 2-3/10-12/14-16 of the oestrous cycle; breed, Large White/Meishan;*, interaction. (DOCX) [file pone.0297875.s003.docx]

***Supplementary Table*** ***2.*** The effect of main factors and their interaction on mRNA and protein expression of ITLN1 in the ovarian follicles as well as its concentration in FF.

|  | **FACTORS** | **F** | **p** |
| --- | --- | --- | --- |
| **ITLN1 GENE**  **EXPRESSION** | phase | F_(2,38)_ = 200.3 | < 0.0001 |
|  | breed | F_(1,38)_ = 349.80 | < 0.0001 |
|  | phase*breed | F_(2,38)_ = 141.70 | < 0.0001 |
| **ITLN1**  **PROTEIN**  **EXPRESSION** | phase | F_(2,24)_ = 1714 | < 0.0001 |
|  | breed | F_(1,24)_ = 1530 | < 0.0001 |
|  | phase*breed | F_(2,23)_ = 192.4 | < 0.0001 |
| **ITLN1 CONCENTRATION IN FF** | phase | F_(2,29)_ = 27.13 | < 0.0001 |
|  | breed | F_(1,29)_ = 18.49 | 0.0002 |
|  | phase*breed | F_(2,29)_ = 1.750 | 0.1916 |
| **EFFECT OF GONADOTROPINS (100 NG/ML) ON ITLN1 PROTEIN EXPRESSION** | hormone | F_(2,17)_ = 273.2 | < 0.0001 |
|  | breed | F_(2,17)_ = 570.4 | < 0.0001 |
|  | hormone*breed | F_(2,17)_ = 273.2 | < 0.0001 |
| **EFFECT OF GONADOTROPINS (100 NG/ML) ON ITLN1 CONCENTRATION IN THE CULTURE MEDIUM** | hormone | F_(2,25)_ = 7.372 | 0.0030 |
|  | breed | F_(1,25)_ = 34.99 | < 0.0001 |
|  | hormone*breed | F_(2,25)_ = 7.930 | 0.0022 |
| **EFFECT OF STEROID HORMONES  (100 nM) ON ITLN1 PROTEIN EXPRESSION** | hormone | F_(3,29)_ = 6.108 | 0.0024 |
|  | breed | F_(1,29)_ = 36.67 | < 0.0001 |
|  | hormone*breed | F_(3,29)_ = 9.438 | 0.0002 |
| **EFFECT OF STEROID HORMONES  (100 nM) ON ITLN1 CONCENTRATION IN THE CULTURE MEDIUM** | hormone | F_(3,19)_ = 283.6 | < 0.0001 |
|  | breed | F_(1,19)_ = 380.8 | < 0.0001 |
|  | hormone*breed | F_(3,19)_ = 19.63 | < 0.0001 |

Abbreviation: FF, follicular fluid; ITLN1, intelectin; phase, 2-3/10-12/14-16 of the oestrous cycle; breed, Large White/Meishan;*, interaction.
