# Supplementary material for: Omentin expression in the ovarian follicles of Large White and Meishan sows during the oestrous cycle and in vitro effect of gonadotropins and steroids on its level: Role of ERK1/2 and PI3K signaling pathways
Source: PLoS One. 2024 Feb 26;19(2):e0297875. doi: 10.1371/journal.pone.0297875 (PMC10896505; doi:10.1371/journal.pone.0297875)
Supplement: S3 Table — Abbreviation: E2, 17β-estradiol; P4, progesterone. The hormone concentrations were evaluated using ELISA. Results are presented as at least eight independent replicates as means ± SEM for each group. Statistical significance is indicated by different letters (p < 0.05). (DOCX) [file pone.0297875.s004.docx]

| ***Supplementary Table 3.*** Concentration of P_4_, E_2_ in plasma and E_2_ in follicular fluid of Large White and Meishan pigs during the oestrous cycle. | | | | | | |
| --- | --- | --- | --- | --- | --- | --- |
| **Breed** | **Large White** | | | **Meishan** | | |
| **Days  of oestrous cycle** | **2 - 3** | **10 - 12** | **14 - 16** | **2 - 3** | **10 - 12** | **14 - 16** |
| **Plasma levels  of P_4_ (ng/ml)** | 0.809 ± 0.185*^aA^* | 3.728 ± 1.164*^bB/C^* | 2.472 ± 0.285*^bB^* | 4.151 ± 1.189*^bC^* | 8.310 ± 1.392*^cD^* | 0.553 ± 0.198*^aA^* |
| **Plasma levels  of E_2_ (pg/ml)** | 15.760 ± 1.375*^bB^* | 7.863 ± 1.487*^aA^* | 9.237 ± 1.992*^aA/B^* | 13.840 ± 1.893*^aB^* | 11.206 ± 1.920*^aB^* | 15.446 ± 2.977*^aB^* |
| **Concentration of E_2_ in the follicular fluid (pg/ml)** | 472.150 ± 10.125*^aA^* | 518.46 ± 12.356*^bB^* | 1897.356 ± 141.526*^cD^* | 521.173 ± 12.426*^aB^* | 1104.153 ± 98.124*^bC^* | 5516.129 ± 123.157*^cE^* |
| Abbreviation: E_2_, 17β-estradiol; P_4_, progesterone. The hormone concentrations were evaluated using ELISA. Results are presented as at least eight independent replicates as means ± SEM for each group. Statistical significance is indicated by different letters (p < 0.05). | | | | | | |
