# Supplementary material for: Omentin expression in the ovarian follicles of Large White and Meishan sows during the oestrous cycle and in vitro effect of gonadotropins and steroids on its level: Role of ERK1/2 and PI3K signaling pathways
Source: PLoS One. 2024 Feb 26;19(2):e0297875. doi: 10.1371/journal.pone.0297875 (PMC10896505; doi:10.1371/journal.pone.0297875)
Supplement: S1 Graphical abstract — (PDF) [file pone.0297875.s005.pdf]

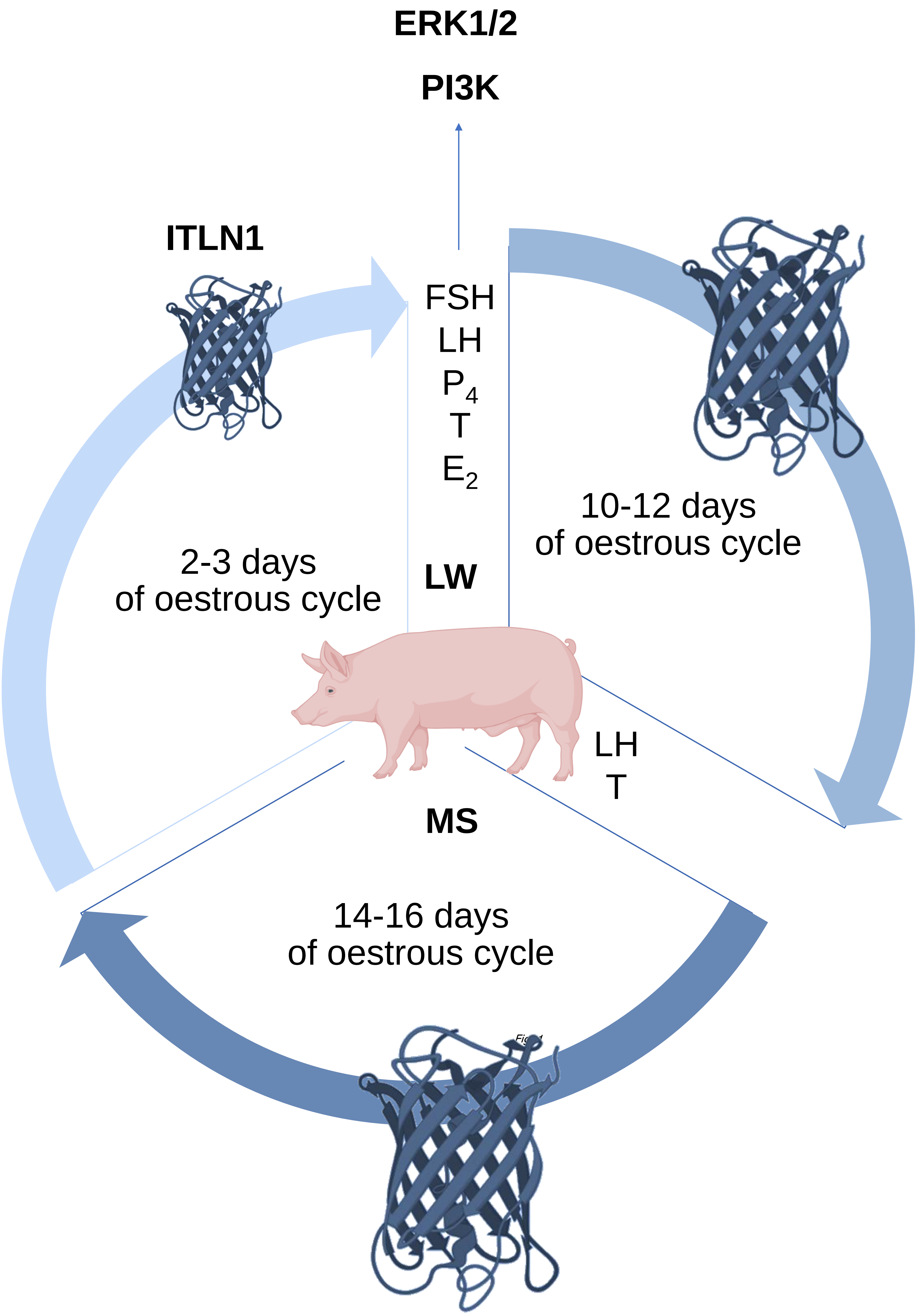

1) The expression (mRNA and protein) of ITLN1 in the ovarian follicles and its concentration in the FF increases with the progression of the oestrous cycle in LW and MS pigs (LW > MS)

2) ITLN1 protein expression, as well as its concentration in culture medium from ovarian follicle cells was increased by FSH, LH, and P_4, E_2, T in LW, while in MS pigs we observed only the stimulatory effect of LH and T

3) ERK1/2 as well as PI3K are associated in the regulation of ITLN1 levels in the cultured ovarian cells

The level of ITLN1 in the ovary depends on the phase of the oestrous cycle and hormones involved in the progression of the oestrous cycle with the participation of ERK1/2 and PI3K kinases
